# Genomic Evolution of the Pathogenic *Wolbachia* Strain, *w*MelPop

**DOI:** 10.1093/gbe/evt169

**Published:** 2013-11-04

**Authors:** Megan Woolfit, Iñaki Iturbe-Ormaetxe, Jeremy C. Brownlie, Thomas Walker, Markus Riegler, Andrei Seleznev, Jean Popovici, Edwige Rancès, Bryan A. Wee, Jennifer Pavlides, Mitchell J. Sullivan, Scott A. Beatson, Amanda Lane, Manpreet Sidhu, Conor J. McMeniman, Elizabeth A. McGraw, Scott L. O’Neill

**Affiliations:** ^1^School of Biological Sciences, Monash University, Clayton, Victoria, Australia; ^2^School of Biological Sciences, The University of Queensland, Brisbane, Australia; ^3^School of Biomolecular and Physical Sciences, Griffith University, Brisbane, Queensland, Australia; ^4^Hawkesbury Institute for the Environment, University of Western Sydney, Penrith, New South Wales, Australia; ^5^Institut Pasteur de la Guyane, Cayenne, French Guiana, France; ^6^Laboratoire des Substances Naturelles Amazoniennes, UMR ECOFOG, Cayenne, French Guiana, France; ^7^Australian Infectious Diseases Research Centre and School of Chemistry and Molecular Biosciences, The University of Queensland, Brisbane, Australia; ^8^Faculty of Veterinary Science, University of Sydney, New South Wales, Australia; ^9^Laboratory of Neurogenetics and Behavior, The Rockefeller University; ^10^Institute for Molecular Bioscience, The University of Queensland, Brisbane, Australia

**Keywords:** *Wolbachia*, evolution, endosymbiont, genomics

## Abstract

Most strains of the widespread endosymbiotic bacterium *Wolbachia pipientis* are benign or behave as reproductive parasites. The pathogenic strain *w*MelPop is a striking exception, however: it overreplicates in its insect hosts and causes severe life shortening. The mechanism of this pathogenesis is currently unknown. We have sequenced the genomes of three variants of *w*MelPop and of the closely related nonpathogenic strain *w*MelCS. We show that the genomes of *w*MelCS and *w*MelPop appear to be identical in the nonrepeat regions of the genome and differ detectably only by the triplication of a 19-kb region that is unlikely to be associated with life shortening, demonstrating that dramatic differences in the host phenotype caused by this endosymbiont may be the result of only minor genetic changes. We also compare the genomes of the original *w*MelPop strain from *Drosophila melanogaster* and two sequential derivatives, *w*MelPop-CLA and *w*MelPop-PGYP. To develop *w*MelPop as a novel biocontrol agent, it was first transinfected into and passaged in mosquito cell lines for approximately 3.5 years, generating *w*MelPop-CLA. This cell line-passaged strain was then transinfected into *Aedes aegypti* mosquitoes, creating wMelPop-PGYP, which was sequenced after 4 years in the insect host. We observe a rapid burst of genomic changes during cell line passaging, but no further mutations were detected after transinfection into mosquitoes, indicating either that host preadaptation had occurred in cell lines, that cell lines are a more selectively permissive environment than animal hosts, or both. Our results provide valuable data on the rates of genomic and phenotypic change in *Wolbachia* associated with host shifts over short time scales.

## Introduction

*Wolbachia pipientis* is an endosymbiotic α-Proteobacterium that infects a broad range of invertebrate taxa, including 40–65% of insect species (Hilgenboecker et al. 2008; Zug and Hammerstein 2012). *Wolbachia* are maternally transmitted, and many insect-infecting strains manipulate their host’s reproductive systems to increase the proportion of *Wolbachia*-infected hosts within a population. The most commonly observed manipulation is cytoplasmic incompatibility (CI), which provides a reproductive advantage to *Wolbachia*-infected females at the expense of their uninfected counterparts (Werren et al. 2008). *Wolbachia* behave as mutualists in filarial nematodes (Smith and Rajan 2000), and there is increasing evidence that they may also benefit at least some insect hosts through metabolic provisioning (Brownlie et al. 2009; Hosokawa et al. 2010) or by protecting their host against pathogens (Hedges et al. 2008; Teixeira et al. 2008; Moreira et al. 2009). Consequently, most relationships between *Wolbachia* strains and their invertebrate hosts range from reproductive parasitism to mutualism. There is, however, an exception to this general trend: the pathogenic *Wolbachia* strain *w*MelPop, also known as *popcorn*.

*w*MelPop was originally identified during a survey of lab lines of *Drosophila melanogaster* for genetic mutations causing brain degeneration (Min and Benzer 1997). As part of an earlier study (Hannah 1949; Valencia and Muller 1949), *D. melanogaster* females that carried recessive alleles of interest on one of their X chromosomes had been crossed with irradiated males carrying the dominant normal alleles. One of the X-chromosome deficiency lines generated by these early studies had a greatly reduced life span when compared with normal flies. Min and Benzer (1997) removed the chromosomal deficiency in this line by crossing with the *white* mutant *w^1118^* and demonstrated that the life-shortening phenotype was caused by a strain of *Wolbachia*. This strain overreplicates in host cells, causing cellular damage and reducing lifespan by approximately one-half in flies (Min and Benzer 1997; McMeniman et al. 2008) and causes similar host effects when transinfected into the mosquito *Aedes aegypti* (McMeniman et al. 2009).

The life-shortening effect of *w*MelPop is being utilized as part of a novel biocontrol strategy to reduce dengue virus transmission by *A. aegypti* (Hoffmann et al. 2011; Walker et al. 2011; McGraw and O’Neill 2013). Like many vector-borne pathogens, dengue requires a period of development within the mosquito vector before it can be transmitted to a new human host; this means that only older mosquitoes are able to transmit dengue. If a mosquito population were infected with *w*MelPop, older mosquitoes would be selectively removed from the population, thus substantially reducing pathogen transmission (Sinkins et al. 1997; Rasgon et al. 2003; Cook et al. 2008). Like a number of other strains of *Wolbachia* (Osborne et al. 2009; Walker et al. 2011; Andrews et al. 2012), *w*MelPop can also interfere with the replication of viruses and other pathogens (Moreira et al. 2009). These abilities of *w*MelPop—to invade mosquito populations through CI, reduce the proportion of older mosquitoes in the population responsible for the majority of disease transmission, and inhibit dengue replication within mosquitoes—make this *Wolbachia* strain a promising tool for the control of vector borne disease in a novel and self-sustaining fashion. Field releases of *w*MelPop-infected *A. aegypti* are currently underway in Australia and Vietnam, and are being planned for additional countries (McGraw and O’Neill 2013).

In addition to its practical applications, *w*MelPop presents a valuable system in which to investigate the relationship between genotype and phenotype in *Wolbachia*. We currently have no genetic transformation capability in *Wolbachia*, and this severely limits functional genetic approaches for investigating the bases of *Wolbachia* strains’ diverse effects on their hosts. Sequencing the genomes of *w*MelPop and related strains offers a potential solution to this problem, for the following two reasons.

First, *w*MelPop is part of a complex of three closely related but phenotypically different *Wolbachia* strains found in *D. melanogaster*. *w*Mel, the genome sequence of which has previously been published (Wu et al. 2004), is the most commonly found strain in global *D. melanogaster* populations today. It is thought to have invaded these populations at some time within the last several thousand years, largely but incompletely replacing the earlier strain *w*MelCS (Riegler et al. 2005; Richardson et al. 2012). Both *w*Mel and *w*MelCS are benign, causing no pathogenesis, and providing strong pathogen blocking (Hedges et al. 2008; Teixeira et al. 2008). Previous work has suggested that the genomes of *w*Mel, *w*MelCS, and *w*MelPop are very similar in structure and in sequence for several genes that have been examined (Sun et al. 2001; Paraskevopoulos et al. 2006). A comparison of the complete genome sequences of these closely related strains could allow us to identify a relatively small number of genomic differences potentially underlying the dramatic phenotypic differences between the benign *w*Mel and *w*MelCS and the pathogenic *w*MelPop.

**Fig. 1.—:**
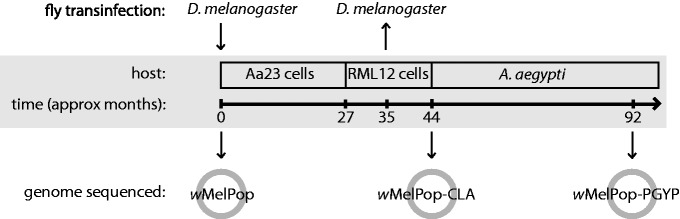
Timeline of the history of the *w*MelPop strains described in this article. The *Wolbachia* strain *w*MelPop was purified from *Drosophila melanogaster w^1118^* and transinfected into the *Aedes albopictus*-derived cell line Aa23. After approximately 27 months of serial passaging, the *Wolbachia* infection was transferred to the RML12 cell line and passaged for a further 17 months, then transinfected into *A. aegypti* mosquitoes. This strain was also transinfected back into *D. melanogaster w^1118^* after approximately 35 months of cell-line passage; this strain, *w*MelPop-CLA, showed reduced pathogenesis in flies compared with the original *w*MelPop strain. We sequenced the genomes of three variants of popcorn: *w*MelPop from *D. melanogaster w^1118^*, *w*MelPop-CLA after approximately 44 months of cell-line passage, and *w*MelPop-PGYP from *A. aegypti* approximately 48 months after transinfection into the mosquito.

Second, to facilitate the transfer of *w*MelPop from *D. melanogaster* to *A. aegypti*, this strain was purified from flies, transinfected into mosquito-derived cell lines and serially passaged for approximately 3½ years before being transferred to mosquitoes (McMeniman et al. 2008, 2009). To determine whether this period of passaging in a novel cellular environment had affected the phenotypes induced by *popcorn*, the endosymbiont was also transferred from cell lines back into *w^1118^* flies and phenotypically recharacterized (fig. 1). After this period of serial passaging in cell lines, *popcorn* remained pathogenic in flies, but to a lesser degree: It grew to a lower density and caused a reduced degree of life shortening and CI (McMeniman et al. 2009). Sequencing and comparison of the genomes of *w*MelPop and the cell-line passaged strain *w*MelPop-CLA could also allow us to identify mutations that could have occurred in these 3 years and may be associated with the observed phenotypic changes.

Here, we describe the draft genome sequences of three sequential variants of *popcorn*: the original *w*MelPop strain from *w^1118^* flies; *w*MelPop-CLA, the strain produced after 3½ years of serial passage in mosquito cell lines; and *w*MelPop-PGYP, purified from *A. aegypti* 4 years after transinfection with *w*MelPop-CLA (fig. 1). We have also obtained genome sequence data of *w*MelCS. By comparing these genomes, we have determined the evolutionary relationships between *w*Mel, *w*MelCS, and *w*MelPop, and identified genomic differences between them. We have also characterized the genetic changes that have occurred during the short period of time during which *w*MelPop was serially passaged in cell lines and have determined the timings of their origin and fixation.

## Materials and Methods

### Cell Lines and Maintenance of *Wolbachia* in Cell Culture

This study used two mosquito cell lines that were infected by the *Wolbachia* strain *w*MelPop and maintained with continuous passaging for several years (McMeniman et al. 2008). Briefly, *w*MelPop bacteria were purified from *Drosophila melanogaster w^1118^* embryos (Min and Benzer 1997; Dobson et al. 2002) and established in a *Wolbachia*-free *A. albopictus* mosquito cell line, Aa23-T (O’Neill et al. 1997). *w*MelPop was maintained in Aa23 cells for a period of approximately 27 months before purification and subsequent introduction into a second mosquito cell line, RML12, also derived from *A*. *albopictus* (McMeniman et al. 2008). The *w*MelPop-infected RML12 cell lines were maintained for a period of 17 months prior to purification and DNA extraction for genome sequencing. Throughout the 44 months that *w*MelPop was maintained in cell culture, insect cells were passaged every 3–4 days and approximately 20% of the cells were used to establish the next generation. Throughout this time, aliquots of *Wolbachia*-infected cells were collected and stored in liquid nitrogen.

### DNA Purification of *w*MelPop-CLA from Cell Culture and Mosquitoes

For *w*MelCS, *w*MelPop, and *w*MelPop-PGYP, purification procedures were followed as described in Iturbe-Ormaetxe et al. (2011). Note that *w*MelPop-PGYP is referred to in that paper as “*w*MelPop-CLA from *A. aegypti* PGYP1.” *w*MelPop-CLA was purified from cell lines as follows. To obtain enough material for the purification of *Wolbachia* DNA from cell lines, 20–30 175 cm^2^ flasks containing confluent monolayers of cells were harvested after gently shaking the bottles. Cells were centrifuged in 50-ml conical flasks at 3,200 × g for 10 min at 4 °C. Culture media was discarded and the pellets were washed twice by resuspending them in SPG buffer (0.25 M sucrose, 0.2% BSA, and 10 mM MOPS, pH 7.2) and then centrifuged at 3,200 × g for 10 min at 4 °C. The supernatant containing *Wolbachia* was sequentially filtered through 5-, 2.7-, and 1.2-µm syringe filters, pelleted by centrifugation at 18,000 × g for 20 min, and then resuspended in cold SPG buffer. Six hundred microliters of resuspended *Wolbachia* were carefully layered on top of a discontinuous Percoll gradient composed of 2.7 ml of 10% (v/v), 3 ml of 15%, 2 ml of 35%, and 4 ml of 50% Percoll/SPG (Duplouy et al. 2013). The equivalent of six to eight bottles of cells was used for each gradient tube. Tubes were centrifuged for 1 h at 8,700 rpm in a Beckman Optima-L-80 XP ultracentrifuge using a swinging bucket rotor SW41. Following centrifugation, four bands containing white cellular material were clearly visible in the interphase between the Percoll layers. The material in each band was recovered by removing the liquid above and pipetting the band out sequentially without disrupting the gradient.

For the extraction of DNA from the different bands, 750 µl of cell material was transferred to Eppendorf tubes and treated with DNaseI for 30 min at 37 °C to remove host DNA contamination. After treating the bands with 1 µl RNase-DNase free (Fermentas), *Wolbachia* cells were disrupted by incubation with proteinase K at 56 °C for 30 min. DNA was extracted using phenol/chloroform, precipitated, washed in 70% ethanol, and resuspended in TE or milli Q water (Millipore).

Total DNA was quantified using a nanodrop spectrophotometer. An aliquot containing approximately 500 ng of the obtained DNA was run on an agarose gel to test DNA quality and purity. DNA that was isolated from each of the different bands in the gradient was characterized for the presence of host, mitochondrial and bacterial contamination by polymerase chain reaction (PCR) using specific primers listed in supplementary table S1 (Supplementary Material online). Band 4 contained the highest concentration of *Wolbachia*, very low levels of host nuclear DNA, and no detectable mitochondrial contamination, and this band was therefore chosen for genomic DNA extraction and sequencing.

### Genome Sequencing, Assembly, and Annotation

Initial genome sequencing was performed by the Australian Genome Research Facility. The *w*MelCS genome was sequenced using Illumina, while *w*MelPop, *w*MelPop-CLA, and *w*MelPop-PYGP were sequenced using 454 (Roche) pyrosequencing (see table 1 for details). Subsequent Illumina resequencing of *w*MelPop and *w*MelPop-PGYP was performed by the Ramaciotti Centre, University of New South Wales.

**Table 1:** Sequencing and Assembly Information for the Genomes of the Strains Described in this Article

Strain	Origin of Material	NCBI Bioproject Identifier	Sequence Type	*Wolbachia* Reads	Contigs/Scaffolds in Assembly	Mean Depth
*w*MelPop	*Drosophila melanogaster w^1118^*	PRJNA196671	454 Titanium PE	265,429	13 scaffolds plus 12 unscaffolded contigs	37
			Illumina 250 bp PE	9,017,059	n/a	1,550

*w*MelPop-CLA	*Transinfected Aedes albopictus*-derived cell line RML12	PRJNA213653	454 GS-FLX shotgun	275,027	220 unscaffolded contigs	49

*w*MelPop-PGYP	*Transinfected A. aegypti* PGYP1	PRJNA213650	454 Titanium PE	888,737	10 scaffolds plus 16 unscaffolded contigs	115
			Illumina 250 bp PE	12,066,179	N/A	2,100

*w*MelCS	*D. melanogaster* Canton-S	PRJNA213657	Illumina 75 bp PE	3,107,396	N/A	114

Note.—PE, paired ends. Approximate mean sequencing depth is estimated by mapping the sequence reads to the *w*Mel genome using default mapper settings and calculating mean total per-site coverage. Complete lists of accession numbers for all data types are given in supplementary information (Supplementary Material online).

The *w*MelPop, *w*MelPop-CLA, and *w*MelPop-PGYP genomes were each assembled using Newbler v2.6. The initial assemblies contained a substantial number of homopolymer errors, which we corrected for *w*MelPop and *w*MelPop-PGYP using Illumina sequencing data generated from the same DNA material that was used for the original 454 sequencing. For each genome assembly, we mapped to the assembly the 454 and Illumina reads from that strain. We then used Nesoni (http://www.vicbioinformatics.com/software.nesoni.shtml, last accessed November 19, 2013) to call variants for each mapping. We considered a variant to be evidence of a homopolymeric sequencing error in the assembly if the following two conditions were met: 1) the Illumina reads, which should not be subject to systematic homopolymer errors, were consistent with the variant and inconsistent with the assembly, and 2) there was disagreement about homopolymer length in the 454 reads mapped to the variant site. In these cases, we corrected the variant to match the Illumina data. Once assembly correction was complete, we compared the genomic arrangement of each strain with that of the complete *w*Mel genome using Mauve (Darling et al. 2004).

The corrected *w*MelPop genome assembly was automatically annotated using the NCBI Prokaryotic Genome Annotation Pipeline (PGAAP; http://www.ncbi.nlm.nih.gov/genomes/static/Pipeline.html, last accessed November 19, 2013). We then compared the gene complements of *w*Mel and *w*MelPop by performing reciprocal BlastN analyses of the genes annotated in the two strains.

### Phylogenetic Analysis

To construct a whole-genome phylogeny of *w*Mel, *w*MelCS, and *w*MelPop, we used the corrected *w*MelPop genome assembly, the published *w*Mel genome (Wu et al. 2004), eight of the *w*Mel consensus genomes and the two *w*MelCS consensus genomes called by Richardson et al. (2012), and a consensus genome generated from our *w*MelCS/Canton-S sequencing data using the same method as Richardson et al. (2012). Briefly, we mapped the *w*MelCS reads to the reference *w*Mel genome using BWA (Li and Durbin 2009), then generated a pileup file with minimum and maximum read depths set to 10 and 100, respectively, and converted the resulting fastq file to fasta format. We then used Mauve (Darling et al. 2004) to align the *w*Mel and *w*MelCS genomes to the *w*MelPop assembly, and exported the core genome regions with minimum LCB length of 100. This produced an alignment of 1,136,727 nt. We then inferred a maximum likelihood phylogenetic tree using RAxML (Stamatakis 2006), with a general time reversible model of nucleotide substitution with a gamma model of rate heterogeneity with four rate categories.

### Identification of Sequence Variants between Genomes

We identified sequence differences between each of the genomes analyzed here (*w*Mel, the three *w*MelCS genomes, *w*MelPop, *w*MelPop-CLA, and *w*MelPop-PGYP) using three main techniques:

1. For the three *popcorn* genome assemblies, we used Mauve (Darling et al. 2004) to create pairwise alignments of each draft assembly to the other assemblies and to the *w*Mel genome, and then exported the core alignments using the stripSubsetLCBs script (provided by Mauve developers at http://gel.ahabs.wisc.edu/mauve/snapshots/, last accessed November 19, 2013). We then used custom Perl scripts to identify mismatches in the alignments.
2. To identify variants between the three *popcorn* genomes, and between the three *w*MelCS genomes and the *popcorn* genomes, we mapped the reads of each of the six data sets to each of the *popcorn* assemblies, and called variants as described later.
3. We also performed an indirect comparison between these six data sets by mapping the reads of each data set to the *w*Mel genome, calling variants, and then comparing variant calls across strains.

For the second and third approaches, we called variants using several methods depending on the kind of sequence data available. For 454 data sets, we mapped reads using Newbler and then examined high confidence variant calls. Illumina data sets were mapped and variants called using two complementary approaches, as follows.

First, reads were aligned with BWA (Li and Durbin 2009) using *aln* and *sampe* with default parameters, and duplicates were removed with *rmdup*. SAMtools (Li et al. 2009) *mpileup* (with parameters -C50 -BEA) and *bcftools* (with parameter -D220) were run to call variants and produce VCF output. Variant sites were then filtered for minimum quality of at least ten and read depth between 10 and 220. Variant calling was repeated on the BWA mapping using Freebayes (Garrison and Marth 2012), and the same filtering steps were applied.

Second, variants were called with the Nesoni high-throughput sequencing data analysis toolkit (http://www.vicbioinformatics.com/software.nesoni.shtml, last accessed November 19, 2013), using SHRiMP (David et al. 2011) as the aligner and Freebayes as the caller. *Nesoni samshrimp* was run with default parameters, and *nesoni filter* was run (with parameter −−monogamous no to retain reads mapping to more than one location). Variants were called by first running *nesoni freebayes* with parameters −−depth-limit 220 and −−ploidy 4 and then reducing the ploidy to 1 by running *nesoni vcf-filter* on the VCF file produced by Freebayes.

Variants identified in one strain were also checked in all other strains, both bioinformatically (using the pipelines above and via manual inspection of read alignments) and using Sanger sequencing. By doing this, we hoped to eliminate biases in variant detection caused by differences in sequence quality or depth in different strains.

### Copy Number Variation across the Genome

We searched for large-scale variations in copy number across the genomes by mapping the sequencing reads of each data set to the *w*Mel genome using BWA (for Illumina data) or Newbler (for 454 data). For the coverage plots in figure 3, we calculated mean per-site total coverage (i.e., including assignment of multiply-mapping reads to a random instance of the repeat in the genome) for nonoverlapping 50-nt windows along the genome. Once we had identified the region of coverage variation in these genomes, we then used additional analyses to identify the boundaries of the triplication and deletion more precisely, as described in supplementary information (Supplementary Material online).

To confirm the triplication of the 19-kb region in *w*MelPop and its deletion in *w*MelPop-PGYP, we determined the relative PCR amplification of two unique sequences in that region (sequences spanning gene boundaries WD0512-WD0513 and WD0513-WD0514) compared with the single copy *wsp* gene as a reference. As a control, we also calculated the relative PCR amplification of the single copy gene WD1213 compared to *wsp*. DNA was extracted from *w*MelCS- and *w*MelPop-infected flies and *w*MelPop-PGYP-infected mosquitoes using a DNeasy Blood and Tissue kit (Qiagen), and 10 ng of DNA was used for qPCR amplification using the primers described in supplementary table S1 (Supplementary Material online). The qPCR reaction contained 10 ng DNA, 5 µl of 2X LightCycler 480 Probes Master containing SYBR green (Roche), and 1 µM of each primer in a total volume of 10 µl. Reactions were performed in triplicate in a LightCycler 480 Instrument (Roche) with the following conditions: 95 °C for 5 min, and 45 cycles of 95 °C for 10 s, 60 °C for 15 s, and 72 °C for 1 s. Relative amplification was calculated using the formula E^(Cp *wsp*)/E^(Cp test gene), where E is the qPCR amplification efficiency, Cp the crossing point, and the test gene was WD0512–WD0513, WD0513–WD0514, or WD1213.

### Searching for Other Structural Differences or New Genes

We used a number of approaches to search for structural variants and insertion of novel genetic material in these genomes. First, we inspected the high confidence structural rearrangements predicted by Newbler after mapping each of the *w*MelPop sequencing reads to the *w*Mel genome. Four variants were identified, all of which were already known: the large inversion, the large triplication, duplication of ankyrin repeats in WD0766, and insertion of an IS5 element in WD1310. Second, we searched specifically for evidence of any additional movement of IS5 elements in *w*MelCS or the *popcorn* genomes. We used the IS5 terminal inverted repeat sequence as a blastN query against the sequencing reads from each data set, and identified reads that matched both this repeat sequence and flanking unique sequence. These reads were then mapped to the *w*Mel genome to identify insertion sites of all IS5 elements from each strain.

Finally, we mapped the reads for *w*MelCS, *w*MelPop and *w*MelPop-PGYP to the *w*Mel genome, then used Perl scripts to identify three sets of cases: 1) read pairs that mapped significantly further apart than expected, possibly due to either genomic rearrangement or a deletion in the query genome, 2) read pairs that mapped in an unexpected orientation, possibly indicating genomic rearrangement, and 3) read pairs in which one read mapped to *w*Mel and the other did not, which could be due to insertion of novel genetic material into the query genome. For each set of cases, we mapped the reads in these categories to the *w*Mel genome and identified regions where there was a concentration of mapped reads. Alignments at all potential sites of interest were then inspected manually. No additional structural variants or sites of insertion of novel genetic material were confirmed.

### Timing of Mutations

To determine the time of appearance of the five mutation events detected during cell passage, cell stocks frozen in liquid nitrogen at different times during cell culture were revived and DNA extracted for PCR analysis. The presence of the 57-kb deletion was detected by PCR amplification of four single copy genes in that fragment (WD0511, WD512, WD513, and WD0514). The insertion of an IS5 element between WD0765 and WD0766 was determined by PCR using primers flanking the insertion site, whereas the presence of the 10 bp in WD0413 was confirmed using a PCR primer whose 3′ end sits in the deletion and only amplifies from the wild type sequence (primers listed in supplementary table S1, Supplementary Material online). Following the screening of all the frozen stocks, the PCR bands obtained were confirmed by sequencing (fig. 6).

## Results

### Draft Popcorn Genomes

The draft *w*MelPop genome consists of 13 scaffolds, ranging in size from 2,302 to 547,521 nt, and an additional 12 unscaffolded small contigs between 599 and 1,946 nt (table 1). The draft genome of *w*MelPop-PGYP is of similar completeness, consisting of ten scaffolds (2,300 to 541,636 nt in length) and 16 unscaffolded contigs (503–1,462 nt). The *w*MelPop-CLA genome was sequenced using shotgun rather than paired-end reads, and the assembly consists of 220 unscaffolded contigs, between 504 and 34,297 nt in length. Like other *Wolbachia* genomes, the *popcorn* genomes are strongly AT-biased, with an average of 36% GC content.

We annotated only the *w*MelPop draft genome. The annotation contains 1,111 protein-coding genes. All genes annotated in *w*Mel have orthologs (or paralogs collapsed into a single contig, in the case of repeat genes such as IS5 elements) in the *w*MelPop assembly. A comparison of the *w*MelPop assembly with the *w*Mel genome confirmed three previously described genomic differences between them. First, although the genomes of both *w*Mel and *w*MelPop each have 13 copies of the IS5 mobile element, only 12 of these copies are shared by both strains. An IS5 element present in *w*Mel between genes WD0516 and WD0517 is not present in *w*MelPop; conversely, an IS5 element has been inserted in *w*MelPop into the ortholog of the *w*Mel gene WD1310 (Riegler et al. 2005; Cook et al. 2008). Second, ankyrin repeat domains have been duplicated in the orthologs of WD0550 and WD0766 in *w*MelPop, and there are also differences in repeat number in the variable tandem repeat region VNTR-141 between the two strains (Riegler et al. 2005, 2012). Third, a ∼143 kb region of the genome has been inverted between *w*Mel and *w*MelPop (Sun et al. 2003; Riegler et al. 2005). The inversion encompasses genes orthologous to WD0399 to WD0536, and is flanked by identical inverted repeat sequences, at *w*Mel coordinates 376,721–379,499 and 522,494–525,272. We also identified over 150 novel differences between *w*Mel and *w*MelPop. All variants discussed later were checked using Sanger sequencing in *w*Mel, *w*MelCS, *w*MelPop, *w*MelPop-CLA, and *w*MelPop-PGYP strains.

We identified and confirmed 156 single nucleotide changes or small indels between the annotated *w*Mel genome sequence and the draft *w*MelPop genome (supplementary data table S1, Supplementary Material online). Of these, 112 (104 SNPs and 8 indels) occur within putative coding regions, and 78 of the SNPs result in a coding change. Five frameshift changes were identified. The first, in the ortholog of WD1155, which encodes a hypothetical protein, should result in the production of a slightly longer protein (118 aa in *w*MelPop vs. 101 aa in *w*Mel). Each of the remaining four frameshifts results in the loss of a stop codon, leading to the production of a single gene in *w*MelPop from what are annotated as two contiguous genes in *w*Mel. In each case, the *w*MelPop CDS has full-length blast matches to genes in other *Wolbachia* strains, suggesting that the single-CDS state is ancestral, and these genes have been relatively recently pseudogenized in *w*Mel. These four genes are the orthologs of 1) WD0026–WD0027, encoding a hypothetical protein, 2) WD1043–WD1044, a second hypothetical protein, 3) WD1215–WD1216, encoding a sensor histidine kinase/response regulator, and 4) WD1231–WD1232, encoding the protoheme biosynthesis protein *HemY*.

### Relationship between *w*Mel, *w*MelCS, and *w*MelPop

**Fig. 2.—:**
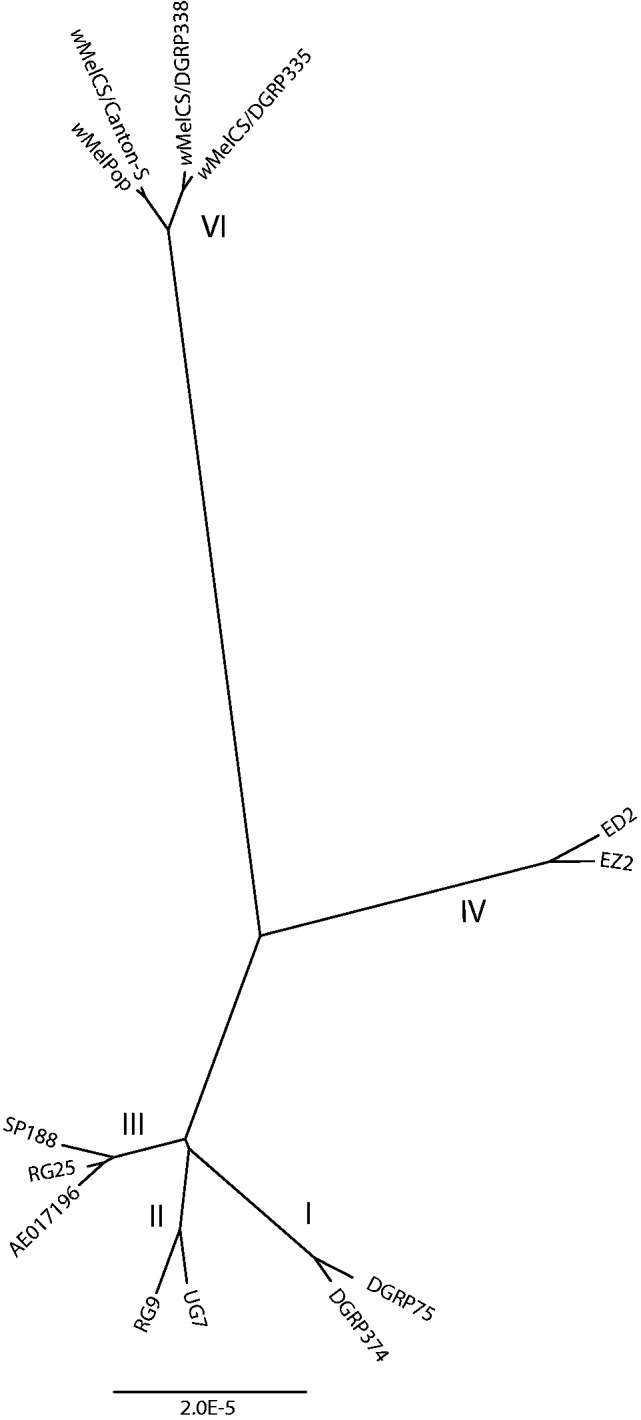
Maximum likelihood phylogeny based on an alignment of the genome sequences of *w*Mel, *w*MelCS, and *w*MelPop strains. The two sequences labeled *w*MelPop and *w*MelPopCS/Canton-S were produced in this article. The other sequences used in the phylogeny are the published *w*Mel genome (Wu et al. 2004), labeled AE017196, and ten consensus genome sequences generated by Richardson et al. (2012); roman numerals indicate the *Wolbachia* clades identified in that paper. *w*MelPop branches within the *w*MelCS clade (VI), separately from the *w*Mel clades (I–IV).

Earlier analyses based on small numbers of sequence differences suggested that *w*Mel and *w*MelPop were sister strains and *w*MelCS a slightly more distant relative (Paraskevopoulos et al. 2006), while analysis of the large inversion, IS5 insertion sites and VNTRs indicated that *w*MelCS and *w*MelPop were more closely related to each other than to *w*Mel (Riegler et al. 2005, 2012). To gain a fuller understanding of the relationship between these strains, we wished to compare their complete genomes. We did not have an assembled genome sequence of *w*MelCS, but we used Illumina sequence data from three *w*MelCS lines. We purified the first of these from a lab line of *D. melanogaster* Canton-S flies (Iturbe-Ormaetxe et al. 2011). The second and third were identified by Richardson et al. (2012) from the sequencing reads of two *D. melanogaster* lines collected from a single population in Raleigh, North Carolina, in 2003 and sequenced as part of the *Drosophila melanogaster* Genetic Reference Panel (Mackay et al. 2012). Richardson et al. (2012) generated a consensus genome sequence for each of these two *w*MelCS lines (and for many additional *w*Mel lines) by mapping the sequence reads from each line to the *w*Mel genome; for consistency we called a consensus genome sequence of our *w*MelCS line using the same technique. We then aligned the published *w*Mel genome, two *w*Mel consensus genomes from each of the four *w*Mel clades identified by Richardson et al. (2012), the three *w*MelCS consensus genomes, and the draft *w*MelPop genome assembly, and constructed a maximum likelihood phylogenetic tree of the sequences (fig. 2).

*w*MelPop clusters clearly with *w*MelCS to the exclusion of all *w*Mel sequences. *w*MelPop and *w*MelCS from Canton-S flies (shown as *w*MelCS/Canton-S) are extremely closely related; the two Raleigh sequences (shown as *w*MelCS/DGRP335 and *w*MelCS/DGRP338) cluster together on a separate branch. This branching pattern indicates that *w*MelPop is recently derived from within *w*MelCS. These inferred relationships are not an artifact of generating the *w*MelCS consensus genome sequences by mapping to the *w*Mel genome: The same patterns of relatedness are observed when *w*MelCS reads are mapped to the *w*MelPop assembly for the purposes of variant calling.

### Genomic Basis of Life-Shortening: Differences between *w*MelCS and *w*MelPop

*w*MelCS is the nonpathogenic strain most closely related to *w*MelPop. This means that the genomic changes that caused *w*MelPop to become pathogenic must have occurred between *w*MelCS and *w*MelPop. To attempt to identify these changes, we mapped the three sets of *w*MelCS reads to the *w*MelPop draft assembly and searched for differences between them. We found a small number of variants between the *w*MelCS/DGRP335 and *w*MelCS/DGRP338 sequences and *w*MelPop (table 2), but at these sites, *w*MelCS/Canton-S always matched the sequence of *w*MelPop, indicating that these differences could not be associated with pathogenesis.

**Table 2:** Eight Sequence Variants Identified between *w*MelPop and the *w*MelCS Isolates Collected in Raleigh in 2003 (*w*MelCS/DGRP335 and *w*MelCS/DGRP338)

*w*Mel Coordinates	*w*Mel	*w*MelCS/DGRP335	*w*MelCS/DGRP338	*w*MelCS/Canton-S	*w*MelPop
12,863	T	C	C	T	T
52,597	TGCGATAAT	TGCGATAAT	TGCGATAAT	—	—
208,096	C	T	T	C	C
297,946	C	C	C	T	T
387,634	G	A	A	G	G
432,815	G	A	A	G	G
1,144,825	TGTTGGTTT	TGTTGGTTT	TGTTGGTTT	—	—
1,254,084	G	A	A	G	G

Note.—In each case, *w*MelCS/Canton-S is identical to *w*MelPop. *w*Mel coordinates and sequences are shown for reference.

The *w*MelPop draft genome assembly contains collapsed repeats and could possibly contain other errors that might impede the detection of sequence variants. To ensure that we were not missing true variants, we therefore also mapped the sequencing reads from each of the three *w*MelCS data sets and the *w*MelPop, *w*MelPop-CLA and *w*MelPop-PGYP data sets against the *w*Mel genome, and compared the variants that were called. We again identified differences between the DGRP *w*MelCS sequences and *w*MelPop (table 2), but no SNPs or indels that could differentiate *w*MelCS/Canton-S from *w*MelPop. We also found no evidence that *w*MelPop and *w*MelCS differ in the insertion sites of IS5 transposable elements or other mobile elements.

**Fig. 3.—:**
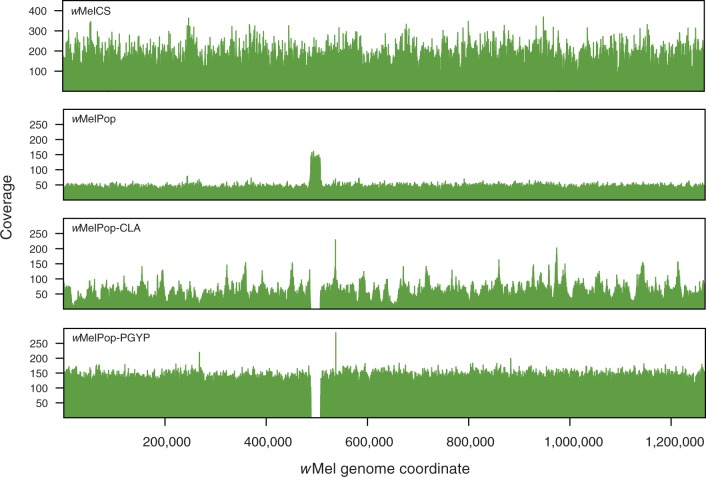
Sequencing coverage for *w*MelCS, *w*MelPop, *w*MelPop-CLA, and *w*MelPop-PGYP. Reads were mapped against the *w*Mel genome using BWA or Newbler with default settings. For depth calculation, reads mapping to repeat regions were assigned to a randomly chosen instance of the repeat, and mean per-site coverage was calculated for nonoverlapping 50-nt windows along the genome. The region corresponding to the genes WD0507–WD0514 in the *w*Mel genome is single copy in *w*MelCS, triplicated in *w*MelPop, and deleted in *w*MelPop-CLA and *w*MelPop-PGYP. The narrow peak of increased coverage visible in *w*MelPop-CLA and *w*MelPop-PGYP slightly downstream of this region represents the duplication of two ankyrin repeats in the orthologs of WD0550 in these strains. This repeat expansion is also present in *w*MelPop (confirmed by PCR), but is not apparent in the sequence coverage plot. Coverage along the genome is clearly more variable for *w*MelCS (100-nt Illumina reads) and *w*Mel-CLA (shotgun 454 reads) than for the two strains sequenced with paired-end 454 reads.

**Fig. 4.—:**
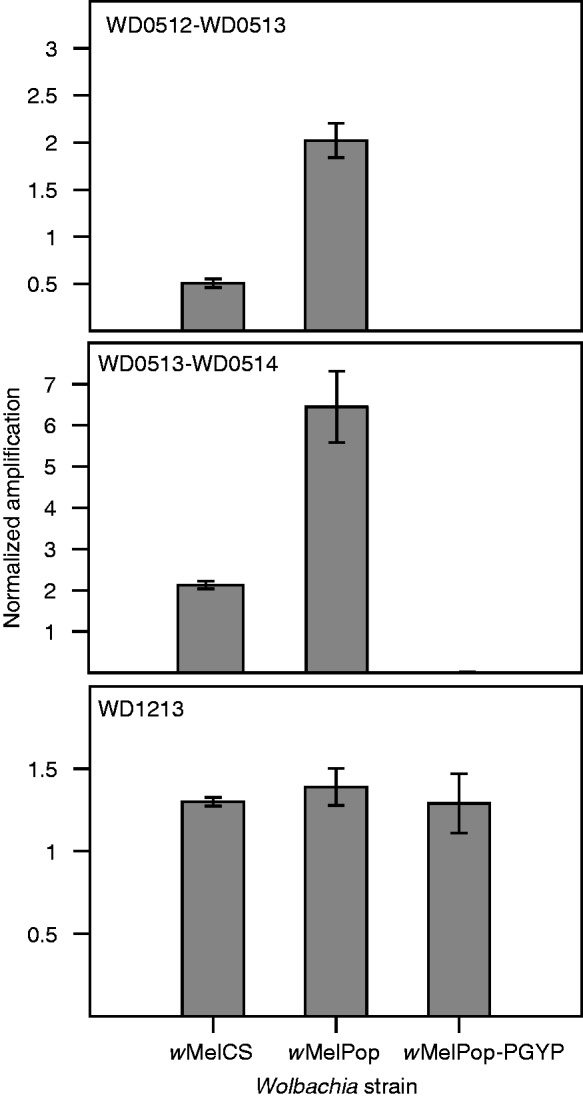
The qPCR analyses showing relative amplification of genes in the putative region of copy number variation (WD0512, WD0513, and WD0514, top two panels) and a control gene outside this region (WD1213, third panel), normalized against the single copy gene *wsp*. For genes WD0512, WD0513, and WD0514, normalized amplification is three times higher in *w*MelPop than in *w*MelCS, whereas there is no amplification in *w*MelPop-PGYP. There are no significant differences in normalized amplification between strains for the control gene WD1213. Note that amplification relative to *wsp* is dependent on primer efficiency, so values on the *y* axis do not represent copy number, and should only be compared across strains, not across genes.

We did, however, detect a region of copy number variation between *w*MelCS/Canton-S and *w*MelPop. A region of the *w*MelPop genome corresponding to the genes WD0507 to WD0514 in *w*Mel has sequence coverage approximately three times higher than that of the rest of the genome (fig. 3; supplementary fig. S1, Supplementary Material online). There is no obvious variation in coverage of any of the *w*MelCS genomes in this approximately 19-kb region. To confirm the triplication of these genes in *w*MelPop, we performed qPCR experiments comparing normalized amplification of genomic DNA from *w*MelCS in Canton-S flies, *w*MelPop in *w^1118^* flies and *w*MelPop-PGYP in *A. aegypti*. Using primers that spanned the gene boundaries WD0512–WD0513 and WD0513–WD0514, and normalizing against the single-copy gene *wsp*, we found that these genes amplify approximately three times as highly in *w*MelPop as in *w*MelCS (fig. 4). These results are consistent with earlier data serendipitously examining expression of these genes using Southern blots (Iturbe-Ormaetxe et al. 2005): in those data, the hybridization signal for these genes, and in particular for WD0514, is stronger in *w*MelPop than in *w*Mel or *w*MelCS, whereas the other genes tested, including *wsp*, produced similar signal intensities for the different strains. It seems unlikely, however, that the triplication of one or all of these genes could be directly responsible for pathogenesis, as the region that is triplicated in *w*MelPop is completely deleted in *w*MelPop-CLA and *w*MelPop-PGYP (confirmed by mapping of sequencing reads to the *w*Mel genome for both substrains, fig. 3 and supplementary fig. S5, Supplementary Material online, and with qPCR for *w*MelPop-PGYP, fig. 4), and yet these two substrains remain pathogenic.

We also tested whether the genetic basis of pathogenesis might be associated with a plasmid present in *w*MelPop but not the nonpathogenic strains. If such a plasmid were present, we would expect the *w*MelPop assembly to contain one or more contigs that do not correspond to matching sequence in *w*Mel. No such contigs were identified in the assembly. We also attempted to separately assemble all reads from the *w*MelPop sequencing run that did not map to the *w*Mel genome, but this produced no large contigs that might form part of a novel plasmid. This is in agreement with earlier laboratory work by Sun et al. (2001) who found no evidence of extrachromosomal DNA on pulse field gel electrophoresis (PFGE) of *Wolbachia* strains including *w*MelPop, indicating that plasmids were not present.

Given the very limited genomic differences we have detected between *w*MelCS and *w*MelPop, we also wished to confirm that the phenotypic differences between *w*MelCS and *w*MelPop are due solely to *Wolbachia*, and not to host effects. *w*MelPop retains its pathogenic phenotype even after being purified and microinjected into *A. aegypti* (McMeniman et al. 2009), demonstrating that the pathogenic effects associated with this strain are clearly caused by *Wolbachia* and are not due to host nuclear or mitochondrial factors. However, it is possible, if unlikely, that *w*MelCS might also have pathogenic capabilities that are suppressed in some way by the Canton-S host background. To test this, we purified *w*MelPop and transinfected it into Canton-S flies that had been antibiotic-treated to remove their native *w*MelCS infection. If Canton-S flies are capable of suppressing pathogenesis, then these flies should live as long as Canton-S flies carrying *w*MelCS. But they do not: female Canton-S flies infected with *w*MelPop (at generation G40 after transinfection) die significantly earlier than Canton-S flies infected with *w*MelCS (median survival 26 days vs. 55.5 days; Cox regression, *χ*^2^ = 4.72, df = 1, *P* = 0.030; see supplementary information [Supplementary Material online] for further details). This confirms that the difference in pathogenicity between *w*MelCS and *w*MelPop is due not to host effects but to *Wolbachia* strain, despite the few genomic differences we have observed between these strains.

### Rapid Evolution of *w*MelPop after Transinfection into Cell Lines

As part of a strategy to attempt to preadapt *w*MelPop derived from *D. melanogaster* before establishing an infection in the mosquito *A. aegypti*, *w*MelPop was passaged in mosquito cell lines for approximately 44 months before being reintroduced to flies and phenotypically recharacterized (McMeniman et al. 2008) (fig. 1). We compared the draft genomes of *w*MelPop and the cell-line passaged strain *w*MelPop-CLA to identify the genetic changes that occurred during evolution in a novel cellular environment, which may be associated with the subsequent attenuation of pathogenesis in *D. melanogaster*. Five genetic differences were detected: an IS5 insertion, a multigene deletion, two point mutations, and a 10-bp deletion.

#### IS5 Element Insertion

**Fig. 5.—:**
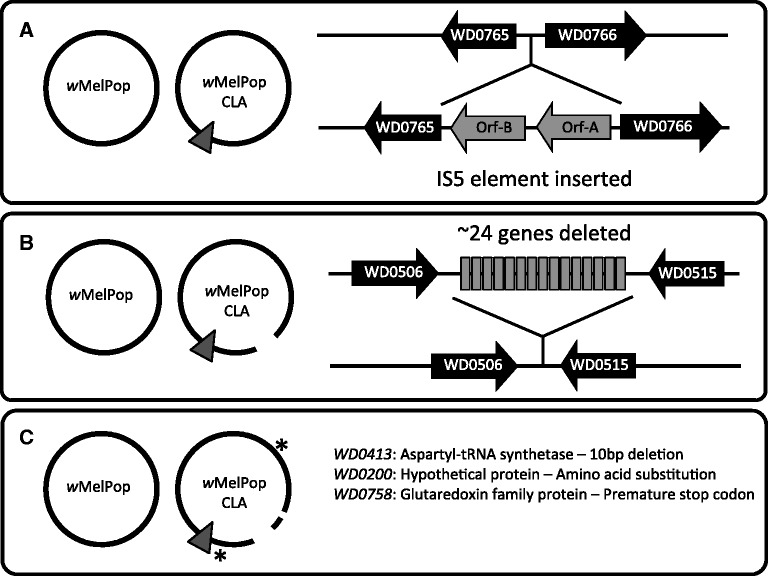
The genomic differences detected between *w*MelPop and the strain *w*MelPop-CLA derived from it through serial passaging in cell lines. (*A*) Insertion of an additional IS5 element between the orthologs of *w*Mel genes WD0765 and WD0766. (*B*) Deletion of a 57-kb region corresponding to the triplicated orthologs of genes WD0507 to WD0514. (*C*) Two point mutations and one 10-nt deletion.

IS5 insertion elements are active and highly polymorphic across different *Wolbachia* strains (Duron et al. 2005; Iturbe-Ormaetxe et al. 2005; Riegler et al. 2005). There are 13 IS5 insertion sequences in the *w*Mel (Wu et al. 2004) and *w*MelPop genomes, 12 of which are common to both strains. *w*MelPop-CLA has an additional copy inserted between the orthologs of genes WD0765 and WD0766, which encode a Na/H+ ion antiporter family protein and an ankyrin domain protein, respectively (fig. 5*A*).

#### Multigene Deletion

A genomic segment homologous to an approximately 19-kb region in *w*Mel has been deleted from *w*MelPop-CLA (fig. 5*B*; supplementary fig. S5, Supplementary Material online). This is the region that is triplicated in *w*MelPop, so the deletion involves the loss of approximately 57 kb of sequence during cell line passaging. PCR-based screens (discussed later) for the presence/absence of single copy genes within this region indicate that the entire segment was deleted as a single event, rather than through gradual genomic erosion. Flanking the deleted region are near-identical retrotransposon sequences that are oriented in the same direction (orthologs of WD0506 and WD0518). A single recombination event between these two sequences may be responsible for the deletion event, consistent with observations in other systems (Gray 2000).

#### Nonsynonymous Mutation in the Ortholog of WD0200

The *w*Mel gene WD0200 encodes a 45 amino acid peptide that is putatively the protein component of RNase P. This ribonuclease cleaves the 5′ leader sequence from precursor tRNA molecules, and may also be involved in preprocessing of other noncoding RNA genes (Ellis and Brown 2010; Krasilnikov 2011). A C-to-T substitution has occurred in *w*MelPop-CLA, which results in the replacement of an aspartic acid for asparagine in the C-terminus of the protein, at amino acid 36 (fig. 5*C*).

#### Frameshift Mutation in the Ortholog of WD0758

WD0758 encodes a 112 amino acid glutaredoxin domain (GRX) protein in *w*Mel. GRX proteins catalyze the reduction of disulfide bonds formed in other proteins and are involved in a diverse range of cellular processes including protein secretion, cell signaling, and DNA replication (Fernandes and Holmgren 2004). Bacterial GRX proteins are also involved in the binding of iron clusters and their delivery to enzymes that use iron. Their mutation can lead to enhanced oxidative stress or decreased growth (Rouhier et al. 2010). The insertion of a G at position 196 results in a frameshift and a premature termination codon, producing a truncated protein that would be 46 residues shorter than the wild-type protein produced by *w*MelPop. As this truncation occurs outside of the GRX domain the effect this mutation would have on the function of WD0758 is unclear; no other GRX domain proteins are thought be encoded by *w*MelPop-CLA.

#### Ten Base Pair Deletion in Ortholog of WD0413

Gene WD0413 encodes a 600 amino acid aspartyl-tRNA synthetase (*aspS*) that facilitates the joining of aspartate to a specific tRNA molecule and is a critical component that maintains translational fidelity (Ibba and Soll 2000). A 10-bp deletion adjacent to the usual stop codon results in a frameshift mutation such that an additional ten amino acids at the C terminus would be incorporated into the protein encoded by *w*MelPop-CLA. A single point mutation close to the C terminus in *E. coli aspS* results in temperature sensitivity and restricts growth at temperatures above 42 °C (Martin et al. 1997). Studies with yeast mutants that have a modification of the last five amino acids of the protein have shown that the C-terminus is involved in acylation and must be folded toward key regions of the enzyme (Prevost et al. 1989).

### Timing of Changes in Cell Lines

During serial passaging, mosquito cells infected with *w*MelPop were passaged every 3–4 days; cells were periodically snap frozen and stored in liquid nitrogen as part of the usual maintenance routine for tissue culture. This collection of frozen cells provided an opportunity to estimate when genetic changes occurred during the evolution of *w*MelPop in mosquito cells. Unique sets of PCR primers (supplementary table S1, Supplementary Material online) were used to screen these banked cells for the mutations discovered in the *w*MelPop-CLA genome.

**Fig. 6.—:**
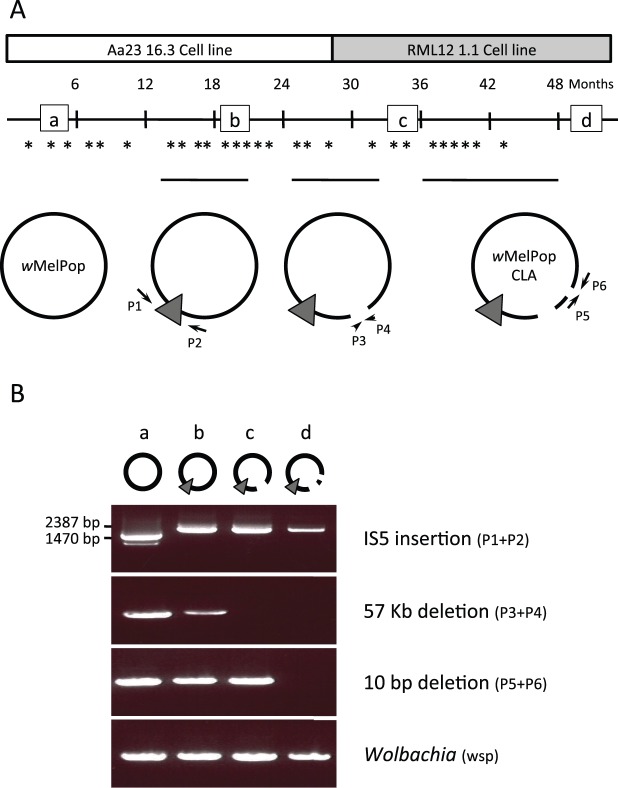
(*A*) Timing of genomic changes during cell line passaging. Asterisks indicate the time points at which PCR assays were performed to test for the presence of each of the three structural genomic changes that occurred. Circular genome icons correspond to the symbols used in figure 5, with arrows labeled P indicating primers used for PCRs. The horizontal black lines above these icons show the period during which each mutation segregated in the population, from first detection to the time at which it was fixed. Small squares labeled a–d indicate time points shown in gel below. (*B*) Ethidium bromide gel showing amplification patterns of these three markers at time points a–d.

The first mutation to occur was the IS5 element insertion between the orthologs of WD0765 and WD0766, which was detected only 13 months after *w*MelPop was established in the Aa23 cell line (fig. 6). For a period of 9 months, two *w*MelPop variants could be detected in the Aa23 cell lines: the wild type (IS5 absent from locus) or the *w*MelPop-CLA form (IS5 present at locus). By 21 months after infection of Aa23, the IS5 insertion at this locus had become fixed within the *Wolbachia* population.

The 57-kb deletion was detectable 25 months after transinfection and was fixed within the *Wolbachia* population 8 months later; during this period, *Wolbachia* was purified from the Aa23 cell line and introduced into a second *A. albopictus* cell line, RML12 (fig. 1). As both the wild type and mutant forms were detected in the early RML12 cell cultures, it is unlikely that the deletion became fixed as a result of a population bottleneck imposed due to *Wolbachia* transinfection into a new insect cell line. Finally, the 10-bp deletion within WD0413 was detectable 37 months after initial cell line infection (10 months after transinfection form Aa23 to RML12), but only became fixed within the population 15 months later.

### Evolution of *w*MelPop-CLA after Transinfection into Mosquitoes

To test whether this rapid rate of genomic change continued after *w*MelPop-CLA was transinfected into mosquitoes, we also purified and sequenced the genome of the *w*MelPop-PGYP strain from mosquitoes (McMeniman et al. 2009) 4 years after the infection was introduced into this host. We compared the *w*MelPop-CLA and *w*MelPop-PGYP genomes by 1) aligning the assemblies to one another and identifying mismatches, 2) mapping the reads of each strain against the assembly of the other strain, and 3) mapping reads of both strains to the *w*Mel genome, then calling and comparing variants. We observed no genetic differences between *w*MelPop-CLA and *w*MelPop-PGYP. We also found no evidence of novel polymorphisms segregating in the population of *w*MelPop-PGYP sequenced.

## Discussion

### Origin of *w*MelPop

The Canton-S line of *D. melanogaster* was collected in Canton, Ohio, prior to 1938 (Bridges and Brehme 1944), and has since been maintained as a common laboratory stock. The *w*MelCS *Wolbachia* strain carried by Canton-S has at least eight genomic differences (table 2) from the *w*MelCS found in the DGRP335 and DGRP338 lines that were collected in 2003 in Raleigh, North Carolina (Mackay et al. 2012). In contrast, we identified only one difference (a change in copy number of a single genomic region) between Canton-S *w*MelCS and the pathogenic *w*MelPop. The original location and date of collection of the line carrying *w*MelPop is unclear, but it had been established in the laboratory prior to 1948 (Hannah 1949; Valencia and Muller 1949). The inferred collection dates of *w*MelPop and *w*MelCS/Canton-S and the extremely close genomic similarity between these two strains suggest that the pathogenic *w*MelPop strain arose from within the *w*MelCS clade at some time in the mid-20th century.

It is possible that the mutation/s that led to *w*MelPop becoming pathogenic occurred in the wild before the line carrying this strain was collected. However, as the fitness costs of *w*MelPop are high, it seems more likely that the evolution of pathogenesis occurred in the laboratory after collection of a line carrying a benign *w*MelCS strain. Although the *D. melanogaster* line carrying the proto-*w*MelPop was crossed with irradiated males of other lines (Hannah 1949; Valencia and Muller 1949), there is no evidence that females of this line were directly exposed to mutagenizing agents. In the absence of paternal inheritance of *Wolbachia*, the mutation/s that led to the development of pathogenesis are likely to have arisen as the result of normal errors in genomic replication, and to have been maintained due to relaxed selection for longevity in a fly stock center environment.

### Genomic Basis of Pathogenesis of *w*MelPop

Despite their dramatic differences in phenotype, we identified only a single genomic difference between *w*MelCS and *w*MelPop: the triplication in copy number of a 19-kb genomic region. This region, which has previously been shown to be highly labile (Iturbe-Ormaetxe et al. 2005; Riegler et al. 2005; Woolfit et al. 2009), contains eight genes, most of which are either transposon-related or annotated as hypothetical proteins. However, it seems unlikely that this increase in copy number is itself associated with pathogenesis, as this same genomic region has been deleted from the pathogenic substrains *w*MelPop-CLA and *w*MelPop-PGYP.

Do the genome sequences of *w*MelCS and *w*MelPop strains differ in other ways that we have not discovered? We know that our data have sufficient power to identify many sequence differences: we found over 150 single nucleotide changes and indels that varied between *w*Mel and *w*MelCS/*w*MelPop. More than 90% of these were independently called as high-confidence variants using sequence data from each of *w*MelCS and *w*MelPop, and all variants that were called in only one data set could later be identified in the sequence reads of the other data set. We were also able to draw on data from three independent sequence data sets for both *w*MelCS and *w*MelPop strains, making it unlikely that stochastic variation in sequence coverage for one genome might be obscuring a genomic difference between them. Nonetheless, there are a number of types of sequence variants that could have remained undetected by our analyses.

First, the genomes of *w*Mel, *w*MelCS, and *w*MelPop are rich in sequence repeats, from short tandem repeat sequences to multiple copies of transposase-related genes each over 2,000-nt long (Wu et al. 2004; Cerveau et al. 2011; Leclercq et al. 2011; Riegler et al. 2012). These repeats present difficulties for both read alignment and de novo assembly (Treangen and Salzberg 2012), and it is possible that sequence variants present in a subset of repeat copies in *w*MelCS and/or *w*MelPop have not been identified. The great majority of *w*Mel annotated repeat sequences longer than 200 nt are associated with mobile elements (Wu et al. 2004), and it seems functionally unlikely that minor sequence variants in one or more copies of these genes might cause pathogenesis. Shorter repeat sequences, however, have been linked to multiple modes of pathogenesis (Delihas 2011; Treangen and Salzberg 2012), and undetected variation in similar sequences in *w*MelPop could be contributing to its pathogenic phenotype.

Second, the fact that numerous *Wolbachia* repeat sequences are longer than the insert size of our paired-end reads means that it is not always possible to assemble or align reads across repeat regions. If long repeats form the breakpoints of structural rearrangements, these events might not be detected. Two observations argue against the possibility that we have overlooked any large genomic rearrangements, however: 1) A large inversion present in the *w*MelPop genome was successfully identified in our mapping analyses, despite being flanked by repeats of moderate length, and 2) an earlier physical and genetic map of *w*MelPop based on restriction endonuclease digestion identified that same inversion as the only large-scale disruption of collinearity between *w*MelPop and *w*Mel (Sun et al. 2003).

Third, although we identified a number of indels in our data sets, indel detection is more challenging and currently less accurate than SNP calling for next-generation sequence data (Albers et al. 2011), and additional indels may have been missed. An indel resulting in a frameshift within a coding gene, or disrupting an intergenic regulatory region, could lead to changes in protein function or expression. Transcriptomic analyses would provide a more direct and powerful way of detecting such changes. Finally, next-generation sequencing read coverage of genomes is nonrandom but shows biases associated with GC content, proximity to the origin of replication and other factors (Minoche et al. 2011; Meglecz et al. 2012), and so some *Wolbachia* genomic regions may be systematically underrepresented in our data. However, when we map the *w*MelCS or *w*MelPop reads against the *w*Mel genome, more than 99% of bases in the reference have better than 10X coverage, which should provide sufficient data to detect variants if present.

It is possible that *w*MelCS and *w*MelPop differ more at the epigenetic than the genomic level. Previous work has shown that bacteria possess diverse adenine methylation systems with a wide range of specificities and activities (Low et al. 2001; Murray et al. 2012), and that changes in this methylation can have large-scale effects on bacterial gene regulation (Fang et al. 2012) and modulate bacterial phenotypes including virulence (Low et al. 2001; Heusipp et al. 2007). The genomes of numerous *Wolbachia* strains are known to encode two phage-derived adenine methylases (Saridaki et al. 2011), and homologs of these genes are present in *w*Mel, *w*MelCS, and *w*MelPop, suggesting that these strains have the genetic machinery required to differentially methylate their genomes. Genome-wide analyses of methylation are becoming increasingly tractable (Murray et al. 2012), and this is a promising avenue for future research on the differences between *w*MelCS and *w*MelPop.

Regardless of potential epigenetic differences between *w*MelCS and *w*MelPop, however, our results demonstrate that endosymbiotic bacteria that differ very little at the genomic sequence level can cause extremely different phenotypes in their hosts.

### Rate of Genomic Change after Transfer to a New Host

*w*MelPop and the *w*MelCS strain from Canton-S flies have been evolving separately for at least 70 years, and yet show very little detectable genetic divergence. This is in strong contrast to the rapid evolution we observed in *w*MelPop after it was transferred into mosquito-derived cell lines. The first mutation, the movement of an IS5 element, occurred within 13 months of transinfection into cells; in contrast, we have identified only two changes in IS5 element location between *w*Mel and *w*MelCS, which diverged several thousand years ago (Richardson et al. 2012). Subsequent genomic deletions and single nucleotide changes occurred within 4 years of the initial transfer to cell lines. This rapid evolution may be due to adaptation to a new host (fly to mosquito), adaptation to the cell line environment, and/or drift due to relaxed selection in cell lines.

There have been relatively few previous studies comparing the complete genome sequences of bacteria before and after transfer to a new host, and most have examined strains or species that have diverged for much longer time periods than the 4 years that separate *w*MelPop and *w*MelPop-CLA. Nonetheless, a number of common patterns associated with bacterial host jumps have emerged. Two independent transfers of *Staphylococcus aureus* from humans to novel hosts have occurred recently: to ruminants within the last 115–1,204 years (Guinane et al. 2010) and to poultry 30–63 years ago (Lowder et al. 2009). In both cases, host adaptation has occurred via a combination of gene loss, acquisition of horizontally transferred genes, and a small-to-moderate amount of diversification in gene sequences. An older transfer, of *Helicobacter* from humans to large felines estimated to have occurred 50,000 to 400,000 years ago, shows the same pattern: host adaptation appears to be primarily driven by change in the accessory gene complement, via pseudogenization, gene deletion, and horizontal gene transfer (Eppinger et al. 2006).

In a study over a more directly comparable timescale, transmission of a single clone of *Escherichia coli* between the members of a family was studied for 3 years (Reeves et al. 2011). Six or more transmission events occurred, including at least two independent interspecies host jumps to the family’s dog. Amongst the 14 isolates sequenced, 20 SNPs were found, but there was no evidence of the movement of mobile elements or gene gain or loss. More of the amino acid changing mutations occurred on the lineages leading to the interspecific transmissions than expected by chance, suggesting that they may be associated with rapid adaptation to the new host.

Our data for *w*MelPop-CLA reflect both gene- and nucleotide-level changes. Deletion of a labile genomic region occurred rapidly, possibly beginning the process of restructuring the accessory genome. No gene gain was observed in *w*MelPop-CLA, as expected in a single-strain laboratory infection. Both gene loss and gene gain, however, are likely to occur in *Wolbachia* strains over longer time periods after host jumps (Duplouy et al. 2013). The 10-bp deletion and two SNPs observed during cell line passaging all affect protein sequence and at least some are likely to have functional consequences, but there are too few changes to robustly conclude that they are due to selective processes.

The fact that we detected no further changes in the genome of *w*MelPop-PGYP after 4 years in mosquitoes might indicate that the burst of substitutions in cell lines reflected adaptation to the mosquito. Alternatively, it may mean that these changes could only be fixed by drift in the permissive cell line environment. In either case, this has implications for the release of *Wolbachia*-infected mosquitoes for biocontrol (McGraw and O’Neill 2013): We do not expect to observe rapid evolution of *w*MelPop-PGYP in released mosquitoes, meaning that pathogen-blocking and life-shortening phenotypes are unlikely to be quickly lost due to changes in *Wolbachia*.

### Use for Functional Genetics

The lack of a genetic transformation technique in *Wolbachia* has inhibited our ability to perform functional genetics on this increasingly important bacterial species. Mutants generated during the maintenance of *Wolbachia* in cell culture for long periods could be isolated and exploited to create novel *Wolbachia* infections in insects or to fine-tune existing transinfected lines using attenuated or virulent variants. Closely related *Wolbachia* variants may also allow comparative genomic studies to link genotype and phenotype as an alternative to genetic transformation, for example, by comparing the phenotypes induced by *Wolbachia* strains before and after deletion, insertion, or mutation events. The five mutations that we identified in *w*MelPop-CLA provide a short list of targets for further functional characterization to investigate potential mechanisms by which *Wolbachia* might adapt to new hosts. Furthermore, using a similar approach to understand the molecular basis for *Wolbachia*-mediated pathogen interference could potentially open new avenues to develop novel antiviral/antimalarial compounds and to identify alternative pathways to target these pathogens.

## Supplementary Material

Supplementary information, table S1, and figures S1–S7 are available at *Genome Biology and Evolution* online (http://www.gbe.oxfordjournals.org/).

Supplementary Data
