# Supplementary material for: Genomic Evolution of the Pathogenic Wolbachia Strain, wMelPop
Source: Genome Biol Evol. 2013 Nov 4;5(11):2189–204. doi: 10.1093/gbe/evt169 (PMC3845649; doi:10.1093/gbe/evt169)
Supplement: Supplementary Data [file supp_5_11_2189__index.html]

Genomic evolution of the pathogenic Wolbachia strain, wMelPop — Genomic Evolution of the Pathogenic Wolbachia Strain, wMelPop — Supplementary Data 

# Genomic Evolution of the Pathogenic *Wolbachia* Strain, *w*MelPop

## Supplementary Data

files

**Files in this Data Supplement:**

- Supplementary Data - pdf file
- Supplementary Data - xls file
