# Supplementary material for: Genomic Evolution of the Pathogenic Wolbachia Strain, wMelPop
Source: Genome Biol Evol. 2013 Nov 4;5(11):2189–204. doi: 10.1093/gbe/evt169 (PMC3845649; doi:10.1093/gbe/evt169)
Supplement: Supplementary Data [file supp_evt169_woolfit.supplementary.pdf]

Supplementary Data

Supplementary Data associated with this paper is divided into two files:

1. Supplementary data file 1 (woolfit.datafile1.xls), which lists the confirmed SNP and indel differences between the genomes of *w*Mel and *w*MelPop.

2. This document (woolfit.supplementary.pdf), which contains the following sections:

1. What are the boundaries of the triplicated region in the wMelPop genome?	p 2
2. What are the boundaries of the large deleted region in the genomes of *w*MelPop-CLA and *w*MelPop-PGYP?	p 8
3. Characterization of *w*MelPop in the Canton-S genetic background.	p 11
4. Supplementary Table 1: Primers referred to in the text	p 20
5. Accession numbers	p 21
6. Supplementary references	p 22

1. What are the boundaries of the triplicated region in the *w*MelPop genome?

It is clear from the coverage graphs shown in Figure 3 in the main text that an approximately 20 Kb region of the genome is triplicated in *w*MelPop. Here we have attempted to estimate the boundaries of the triplicated unit more precisely.

We mapped all *w*MelPop 454 reads to the *w*Mel reference genome, and used Perl scripts to extract coverage information from the Newbler output file 454AlignmentInfo.tsv. We then examined two measures of read mapping coverage around the triplicated area of the *w*MelPop genome (Supplementary Figure S1). *Unique coverage* at a site is the number of non-duplicate, uniquely mapping reads that align at that site. *Total coverage* is calculated as the sum of unique reads mapped to that site plus the estimated number of repeat reads mapped to the site. The repeat estimate is made by assigning each repeat read to a randomly chosen instance of the repeat in the genome.

The genomic region extending from the start of WD0507 to the end of the intergenic region following WD0514 has coverage approximately three times greater than average for the rest of the genome (Figure S1, top panel). It is not clear, however, whether the repeat genes flanking this region - WD0506 at the 5' end and WD0515 and WD0518 at the 3' end - are also part of the triplicated unit. Their estimated coverage is intermediate between that of the triplicated region and the rest of the genome (Figure S1, second panel), but as any increase in reads matching these repeats due to triplication will be randomly distributed across all repeat copies in the reference genome, an increase in copy

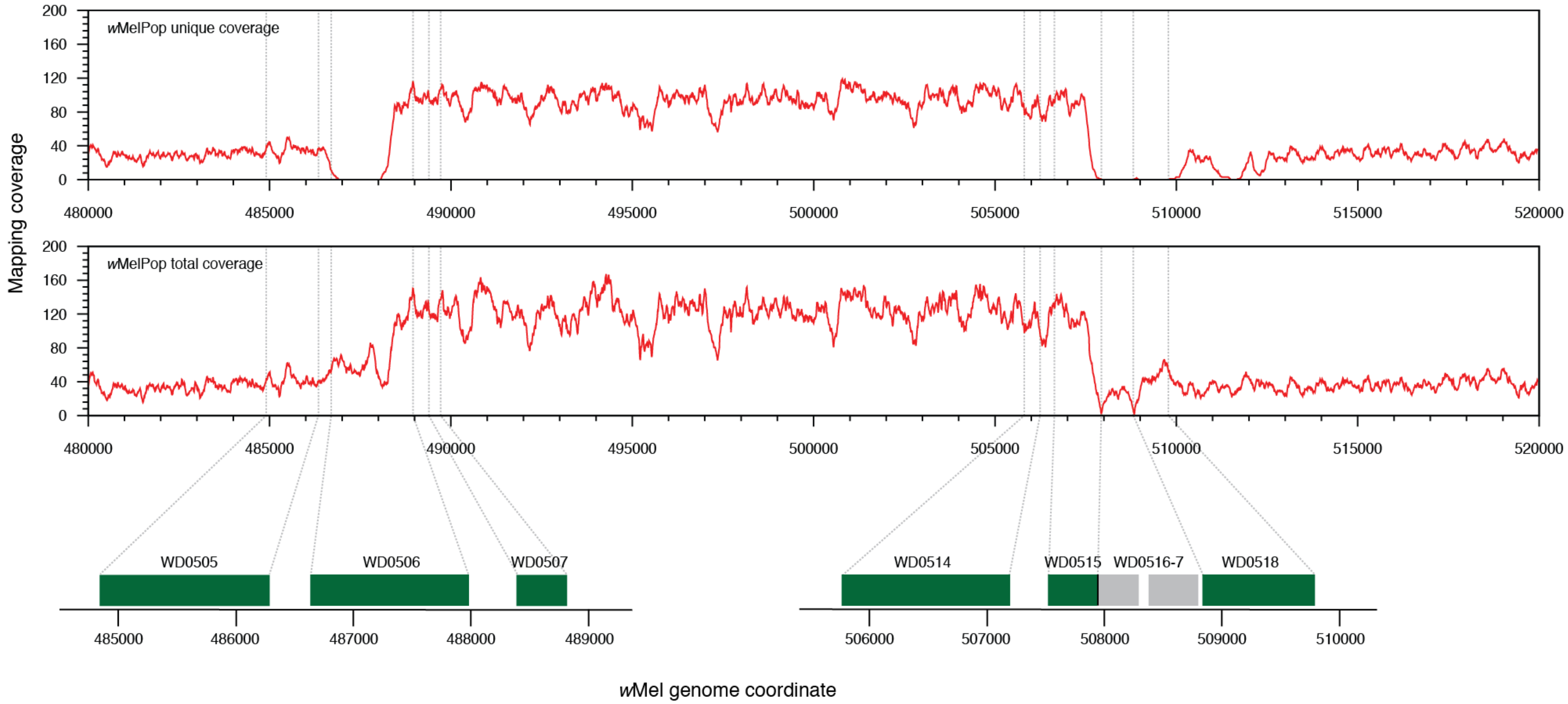

Figure S1: Coverage depths of *w*MelPop 454 reads mapped to the *w*Mel genome between coordinates 480,000 and 520,000. Two plots are shown: unique coverage (top panel) and total coverage (second panel). The third panel shows *w*Mel genes surrounding the boundaries of the triplicated region in green; the copy of the IS5 element encoded by WD0516 and WD0517 in *w*Mel is absent from the *w*MelPop genome so these genes are shown in grey.

number would be difficult to detect, given the inherent random variation in coverage along the genome. We therefore took a different approach to test whether these flanking repeat genes are part of the triplicated unit, by attempting to estimate the distance separating the known portions of the units.

In the schematic below, each green block represents the unit that is known to be triplicated, which extends from the start of WD0507 (marked A_i) to the end of the intergenic region following WD0514 (marked B_i). The flanking repeat regions are represented as blocks of light orange (WD0506) and dark orange (WD0515 and WD0518). If only the region A to B is triplicated, then at each of the junctions between the triplicated units the gap between the B at the end of one unit (e.g. B_1) and the A at the start of the subsequent unit (e.g. A_2) will be 0 nt in length (Figure S2).

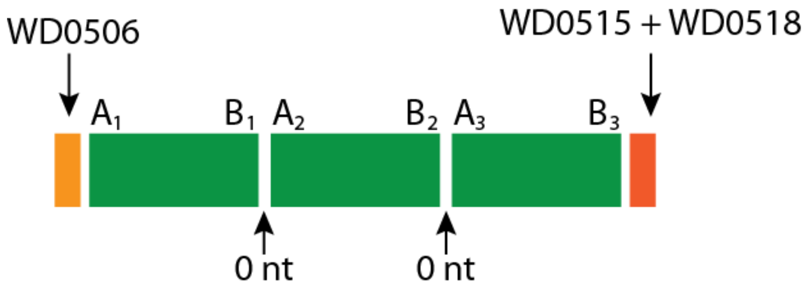

Figure S2

On the other hand, if both sets of flanking repeat sequences are also part of the triplicated unit, then the gap between B_1 and A_2 may be as long as 2700 nt (Figure S3).

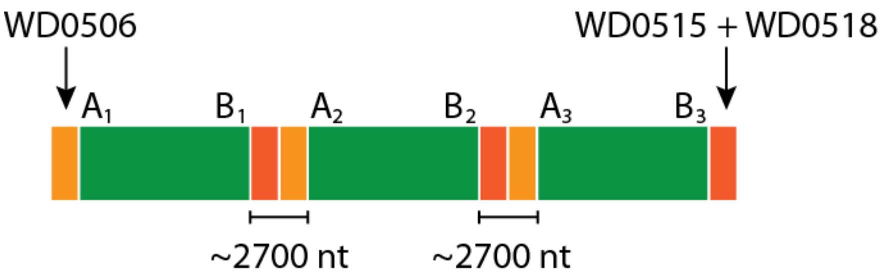

Figure S3

To try and estimate the length of this gap, and thereby infer whether some, all or none of the flanking repeat sequences are included in the triplicated unit, we identified read pairs that spanned the junction between units. We used Perl scripts to scan the Newbler output file 454PairAlign.txt and identify paired-end reads which matched four criteria: (1) each read in the pair could be mapped to a unique location in the *w*Mel reference genome, (2) the mapping location of one read was within 4000 nt of start of the known triplicated unit (i.e. between *w*Mel coordinates 488,397 and 492,397), (3) the mapping location of the other read was within 4000 nt of the end of the known triplicated unit (i.e. between *w*Mel coordinates 503,580 and 507,580), and (4) the direction of mapping of the reads confirmed that the reads mapped across a junction of the tandem repeat unit, rather than within a single unit. We identified 46 read pairs that met these criteria.

The average distance between properly mapped read pairs for the complete *w*MelPop dataset is 1923 nt, with a range of 954 to 3872 nt (from Newbler output file 454NewblerMetrics.txt). We used this information, and the mapping locations of the 46 repeat pairs described above, to estimate the length of the gap between B_1 and A_2 (and between B_2 and A_3, assuming the two junctions are identical) as follows.

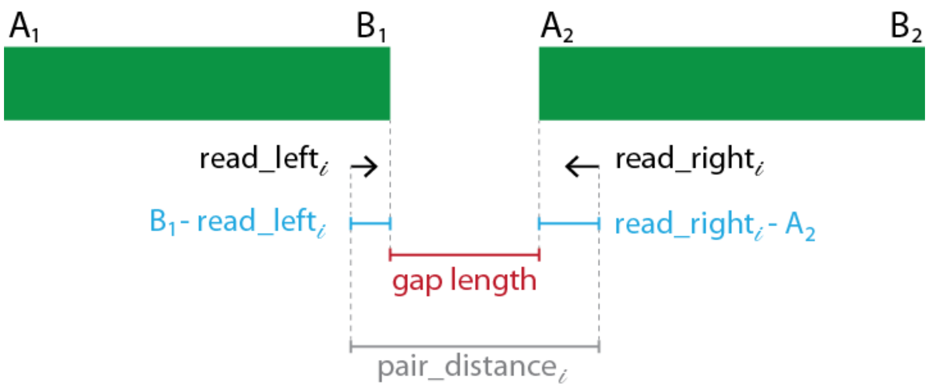

Figure S4.

The distance, pair_distance$_i$ (Figure S4), between the mapping locations of a pair of reads, read_left$_i$ and read_right$_i$, will be the sum of the distance from the start of read_left$_i$ to B_1, the distance from A_2 to the end of read_right$_i$, and the gap between B_1 and A_2:

$$\text{pair\_distance}_i = (B_1 - \text{read\_left}_i) + (\text{read\_right}_i - A_2) + \text{gap length}$$

which can be trivially rearranged to allow us to estimate gap length:

$$\text{gap length} = \text{pair\_distance}_i - (B_1 - \text{read\_left}_i) - (\text{read\_right}_i - A_2)$$

As we know that pair distance varies for individual read pairs, we can obtain a more accurate estimate of the gap length by averaging across all 46 read pairs that span the gap:

$$\text{gap length} = \Sigma\,(\text{pair\_distance}_i - (B_1 - \text{read\_left}_i) - (\text{read\_right}_i - A_2))\;/\;46$$

If we substitute the average known pair distance of 1923 nt into the equation above, we obtain an estimated gap length between B_1 and A_2 of 208 nt. Estimates of gap length for individual read pairs range from -1370 to 1235 nt, and the standard deviation of the mean is 636 nt.

Negative gap length estimates indicate that, as expected, a range of fragment sizes were sequenced in the 454 library, and some were shorter than the mean size of 1923 nt.

Although the error associated with our estimate of the gap length is large, these data do allow us to exclude two possible scenarios for the boundaries of the triplicated unit. If full-length paralogs of both WD0506 and WD0515/WD0518 were part of the triplicated unit, the gap length between B_1 and A_2 would be approximately 2700 nt; if only one of the flanking repeats were included the gap would be a little longer than 1300 nt. Both of these hypotheses can be rejected by our data. We cannot exclude the possibility that the gap between B_1 and A_2 is 0 nt. However, given that the mean estimate is 208 nt, we believe that it is most likely that a partial fragment of the flanking repeat/s is included in the triplicated unit. This is supported by the fact that we were able to identify a number of single reads matching the boundary between B and WD0515, and between WD0506 and A, but no reads matching a boundary between contiguous B_1- A_2 sequences, which should be present if no flanking repeat sequence were included in the triplicated unit.

2. What are the boundaries of the large deleted region in the genomes of *w*MelPop-CLA and *w*MelPop-PGYP?

Figure 3 in the main text makes it clear that approximately the same genomic region that is triplicated in *w*MelPop has been deleted in *w*MelPop-CLA and *w*MelPop-PGYP. Here we attempt to identify the boundaries of this deletion more precisely. We have used sequence data from *w*MelPop-PGYP only, as it is paired-end data while we have only shotgun data from *w*MelPop-CLA. However, based on the coverage plots shown in Figure 3, we expect that the boundaries of the deletion will be the same in both substrains.

We could obtain an approximate estimate the boundaries of the deletion by using the coordinates of the genomic region with unique or total coverage of 0 (Supplementary Figure S5). However, the unique coverage plot is likely to overestimate the size of the deletion if repeat sequences flanking this region are not completely deleted in *w*MelPop-PGYP. In contrast, the total coverage plot may underestimate the size of the deletion if the repeat coverage surrounding the deletion reflects random mapping of shotgun reads that in reality belong to other instances of these repeat genes in the genome.

We therefore sought to identify the boundaries of the deletion based only on the mapping of read pairs that we could be confident were mapped to those instances of the repeat sequences flanking the deletion. We used Perl scripts to parse the Newbler output file 454PairAlign.txt and identify read pairs that matched two criteria:

(1) One read of the pair had a unique mapping location in the *w*Mel genome, and that hit was within 4000 nt of the edge of the deletion as defined by the unique coverage plot (i.e. between

coordinates 482,855 and 486,855, or between 509,878 and 513,878). These were considered "anchor" reads.

(2) The other read of the pair mapped to one or more locations in the *w*Mel genome, and at least one of those hits fell between the edge of the deletion as defined by unique coverage and 4000 nt further into the deletion (i.e. between 486,855 and 490,855 or between 505,878 and 509,878). These were "extension" reads.

We chose 4000 nt as the window size for both these criteria as this is approximately the maximum distance between mapped pairs in the *w*MelPop-PGYP dataset.

At the 5' end of the deletion, we identified anchor reads with mapping coordinates on the *w*Mel genome ranging from 483,015 to 486,514, and extension reads from 487,009 to 488,420. At the 3' end of the deletion, anchor reads mapped from 510,006 to 513,949, while extension reads were from 507,485 to 509,796. Our best estimate of the boundaries of deletion is therefore that it extends from *w*Mel coordinates 488,421 to 507,484. The 5' end of this region is just after the start of the gene WD0507, which has coordinates 488,397-488,804. The 3' end is intergenic between WD0514 (505,791-507,200) and WD0515 (507,580-507,960). We therefore believe that the deletion encompasses the *w*MelPop-PGYP genes homologous to WD0507 to WD0514, inclusive. (Note that these genes are single copy in *w*Mel, but triplicated in *w*MelPop, as described previously.) The genes flanking the deleted region, WD0506 and WD0515, are both pseudogenized reverse transcriptases.

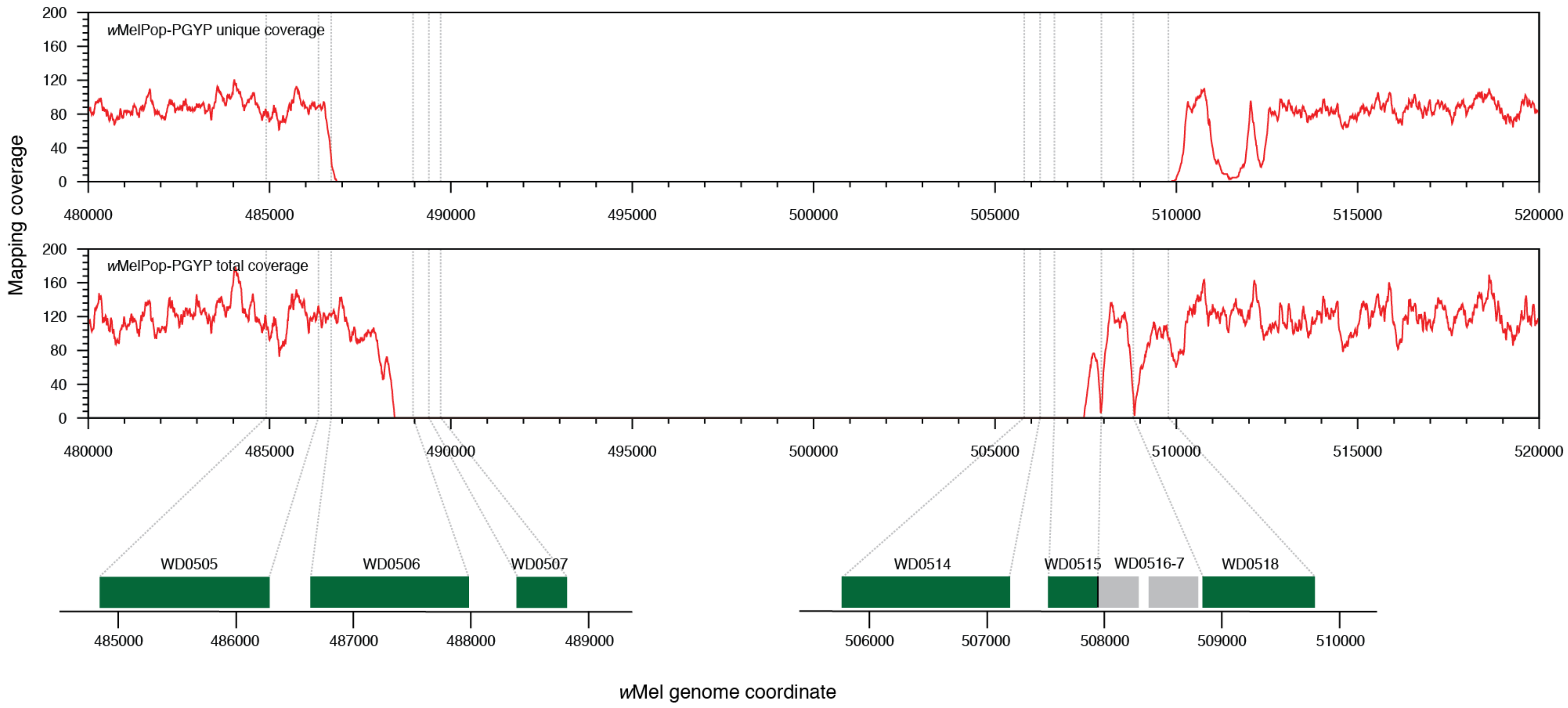

Figure S5. Coverage depths of *w*MelPop-PGYP 454 reads mapped to the *w*Mel genome between coordinates 480,000 and 520,000. Details of plot construction are as for Figure S1, above.

3. Characterization of *w*MelPop in the Canton-S genetic background

To test whether the *D. melanogaster* Canton-S background has the ability to supress the life-shortening phenotype typically induced by the *Wolbachia* strain *w*MelPop, we transinfected *w*MelPop from the w^{1118} line into Canton-S flies. We then compared the density and life-shortening capabilities of the *w*MelPop and *w*MelCS strains in the Canton-S background. The *w*MelPop strain induced the life-shortening phenotype, while the lifespan of Canton-S adult flies naturally infected with the *w*MelCS strain was not significantly different to *Wolbachia*-uninfected flies. Quantitative PCR revealed that the density of the avirulent *w*MelCS strain in young adult Canton-S female flies was significantly higher than *w*MelPop strain. In older flies, the density of the *w*MelPop strain increased significantly to levels approximately 10 fold higher than the *w*MelCS strain. Our results indicate that Canton-S does not repress pathogenesis, and that phenotypic differences between *w*MelCS and *w*MelPop are due to *Wolbachia* strain rather than host effects.

Methods

Embryonic Microinjection. Embryo cytoplasm transfer between *Drosophila* preblastoderm embryos was carried out using a method developed for injection of mosquito embryos [1] that allowed a large amount of cytoplasm (approximately 5%) to be transferred from donor to recipient host. The transfer of embryonic cytoplasm is the most direct route for transinfection with minimal *Wolbachia* mortality. Donor and recipient embryos were collected every 30 min using molasses agar plates with live yeast paste. Embryos still containing an intact chorion were aligned against a thin hydrophilic blotting membrane in contact with moist filter paper. After transfer of approximately 10 donor and 30 recipient embryos,

the membrane was dried with filter paper to slightly dehydrate the embryos. Microinjection was carried out under x100 magnification using a FemtoJet microinjector system (Eppendorf) with type II femtotip microinjection needles (Eppendorf). An Eppendorf Transferman micromanipulator was used to manipulate the microinjection needles. Cytoplasm was withdrawn from the posterior poles of donor embryos and immediately injected into the posterior poles of recipient embryos. After injection, the membrane was removed and paraffin liquid was added to the embryos to prevent further embryo desiccation. All donor and recipient embryos used in the study were younger than 90 min of age to ensure injection of *Wolbachia* prior to pole-cell formation. Injected G0 embryos were monitored 18-36 hours post injection to ensure hatched larvae were immediately transferred to standard cornmeal based *Drosophila* rearing medium and incubated at 24°C. A density of approximately 50 larvae / vial was used to provide optimal rearing conditions.

Screening and selection of transinfected lines. Surviving virgin G0 females resulting from injected embryos were placed in individual vials containing rearing medium and mated to three *Wolbachia*-uninfected males to establish isofemale lines. After egg laying and evidence of viable G1 offspring, DNA was extracted from G0 females using the STE boil method (STE 100 mM NaCl 10 mM Tris HCl, pH 8.0 1 mM EDTA) [2]. PCR screening for the *w*MelPop strain in the G0 generation of transinfected females was carried out using PCR primers specific for the IS5 repeat element: IS5-FWD1 (5'-GTATCCAACAGATCTAAGC) and IS5-REV1 (5'-ATAACCCTACTCATAGCTAG). IS5 is a multi-copy insertion element providing a sensitive but specific target for *w*MelPop infection status [3]. Amplification of DNA was carried out in 20 µL reaction volumes which included: 2.0 µL of 10X buffer (NEB), 25 µM of dNTPs, 0.5 µM of forward and reverse primer, 0.75 U of Taq polymerase (NEB) and 1.0 µL of DNA

template. PCR conditions were as follows: denaturation at 94 °C for 3 min; 35 cycles of denaturation at 94 °C for 30 sec, annealing at 55 °C for 30 sec, and extension at 72 °C for 1 min; followed by a final 10 min extension step at 72 °C. To select for a stable transinfection, offspring from G0 females that were positive for *w*MelPop infection by IS5 PCR screening were used as parental stock. All resulting G1 females were isolated as virgins, placed into individual vials and outcrossed to three *Wolbachia*-uninfected males. The progeny resulting from transinfected G1 females was reared to adulthood and screening and selection was continued until the G6 generation. Lines were closed by allowing the males and females of the G7 generation to mate with one another.

Longevity assays. The adult longevity of CS*w*MelPop (G20 transinfected line), CS*w*MelPop.tet, w^{1118}, Canton-S and Canton-S.tet adult flies were assayed at 26°C. Tetracycline treatment to remove the transinfected *w*MelPop *Wolbachia* and generate a CS*w*MelPop.tet line was carried out by addition of tetracycline (0.3 mg/ml) to the adult diet for two generations and fies were then transferred to a normal diet for five generations prior to lifespan assays. The assay was repeated with females of the G40 post-transinfection CS*w*MelPop line to determine any changes in the life-shortening phenotype. Longevity assays were conducted as previously described [4]. In each assay, 10 sets of 10 flies for each sex were maintained at 26°C in standard cornmeal food vials without additional live yeast. The number of new deaths was recorded each day and living flies were moved to fresh food vials every 5 days. *Drosophila* survival was analysed using Cox regression to determine the equality of the survival distributions between lines after pooling data across replicates.

***Wolbachia* density**. The density of the *w*MelPop strain in the Canton-S background (CS*w*MelPop transinfected line G40) was compared to the *w*MelCS strain in Canton-S flies. For each fly line, 10 sets of 10 virgin female

flies were maintained at 26°C in standard cornmeal food vials without additional live yeast. At days 3,7,14 and 28 post adult emergence, 6 adult flies of each line per time point were transferred to the -80°C freezer. Head dissection was performed on all samples and DNA extraction carried out on heads and the remaining thorax/abdomen as previously described. *Wolbachia* density was assessed by comparing the abundance of the single-copy *Wolbachia* surface protein gene (*wsp*) to that of the single-copy *Drosophila melanogaster rps17* gene. Primers for *Wolbachia* (wspFQALL 5′ GCATTTGGTTAYAAAATGGACGA 3′ and wspRQALL 5′ GGAGTGATAGGCATATCTTCAAT 3′) and for the host gene RPS17 (Dmel.rps17F 5′CACTCCCAGGTGCGTGGTAT 3′ and Dmel.rps17R 5′GGAGACGGCCGGGACGTAGT 3′) are previously described [5]. Reactions were done in duplicate in a Rotor-gene thermal cycler (Corbett Life Sciences) with the following conditions: one cycle of 50°C 2 min, 95°C 2 min, followed by 40 cycles of 95°C 5 sec, 60°C 5 sec, 72°C 10 sec. Ratios were calculated in Qgene and statistical analysis included Mann-Whitney t test to compare differences of the means.

Results

Intraspecific transfer of *w*MelPop into Canton-S background. The *w*MelPop strain was transferred from w^{1118} flies into tetracycline treated Canton-S.tet flies using embryonic cytoplasm transfer. The Canton S.tet line was previously generated by tetracycline treatment to remove the *w*MelCS strain. A total of 176 Canton S.tet embryos injected with *w*MelPop-infected w^{1118} cytoplasm resulted in 7 fertile adult G0 females. 4 of 7 (57%) of these surviving G0 females were infected with the *w*MelPop strain with 3 independently generated G1 lines infected with the *w*MelPop strain. One line, termed CS*w*MelPop, was selected for further characterization and a

tetracycline-cured line (CS*w*MelPop.tet) was established by G8 post-infection.

Life-shortening phenotype. Lifespan assays of adult male and female flies (Figure S6) were carried out at a temperature of 26°C and the G20 and G40 generations of the transinfected CS*w*MelPop line were assayed. For females (Figure S6a), a median survival of 39 days for CS*w*MelPop G20 was significantly greater than the 29 days for w^{1118} females (Cox regression, X^2 = 40.25, df = 1, P < 0.001). However, this median survival was significantly less than Canton-S flies infected with the *w*MelCS strain of *Wolbachia*, 55.5 days, (Cox regression, X^2 = 50.28, df = 1, P < 0.001). The life-shortening capability of the *w*MelPop strain in the Canton-S background increased relatively rapidly and by G40 post infection the median survival of CS*w*MelPop G40 females was only 26 days. The lifespan of Canton-S flies infected with the *w*MelPop strain was significantly less than the naturally infected w^{1118} females (Cox regression, X^2 = 4.72, df = 1, P = 0.030). The median survival for *Wolbachia*-uninfected CS*w*MelPop.tet and Canton S.tet females (56 and 50 days respectively) confirmed that reduced adult longevity is dependent on infection with the *w*MelPop strain (Cox regression, X^2 = 35.1, df = 1, P < 0.001; X^2 = 15.9, df = 1, P < 0.001).

A similar change in the life-shortening phenotype was observed for adult male flies of the transinfected CS*w*MelPop line, as shown in Figure S6b. A median survival of 35 days for CS*w*MelPop G20 males was significantly greater than 27 days for w^{1118} males (Cox regression, X^2 = 38.21, df = 1, P < 0.001). This median survival was, however, significantly less than Canton-S males infected with the *w*MelCS strain (Cox regression, X^2 = 23.87, df = 1, P < 0.001). The median survival of CS*w*MelPop G40 males was only 24 days which was not significantly different to w^{1118} males (Cox regression, X^2 = 2.56, df = 1, P = 0.110). The median survival for *Wolbachia*-uninfected CS*w*MelPop.tet and Canton S.tet males (48.5 and 44 days respectively)

were significantly higher (Cox regression, X^2 = 43.4, df = 1, P < 0.001; X^2 = 27.2, df = 1, P < 0.001).

***Wolbachia* density in adult female flies**. The density of the *w*MelPop and *w*MelCS strains was compared over the adult female lifespan in the Canton-S background to determine if the life-shortening phenotype was correlated to *Wolbachia* density. As shown in Figure S7, surprisingly the density of the avirulent *w*MelCS strain was significantly greater than the *w*MelPop strain in both 3-day-old heads (two-sample t-test: t=9.271, df=10, P<0.0001) and the remaining thorax/abdomens (two-sample t-test: t=10.28, df=10, P<0.0001). The density of the *w*MelCS strain was also significantly greater than the *w*MelPop strain in both 7-day-old heads (two-sample t-test: t=8.148, df=10, P<0.0001) and the remaining thorax/abdomen (two-sample t-test: t=14.92, df=10, P<0.0001). For 14-day-old female flies, the densities of the two *Wolbachia* strains were not significantly different in both heads (two-sample t-test: t=1.541, df=10, P=0.1544) and thorax/abdomens (two-sample t-test: t=0.741, df=10, P=0.4758). However, the density of the *w*MelPop strain was approximately 10 fold higher than the *w*MelCS strain in 28-day-old heads (two-sample t-test: t=6.692, df=10, P<0.0001) and approximately 7 fold higher in 28-day-old thorax/abdomens (two-sample t-test: t=8.262, df=10, P<0.0001).

Figure S6. Effect of the *w*MelPop strain on adult *Drosophila melanogaster* longevity in a) females and b) males. The lifespan of CS*w*MelPop (G20 and G40 generations), w^{1118}, CS*w*MelPop.tet, CS and CS.tet adult flies was assayed at 26°C with 10 replicate vials of 10 flies of each sex. Each day, the number of new deaths was recorded. Flies were moved into fresh food vials every 5 days. *Drosophila* survival was analysed using Cox regression to determine the equality of the survival distributions between lines after pooling data across replicates.

a

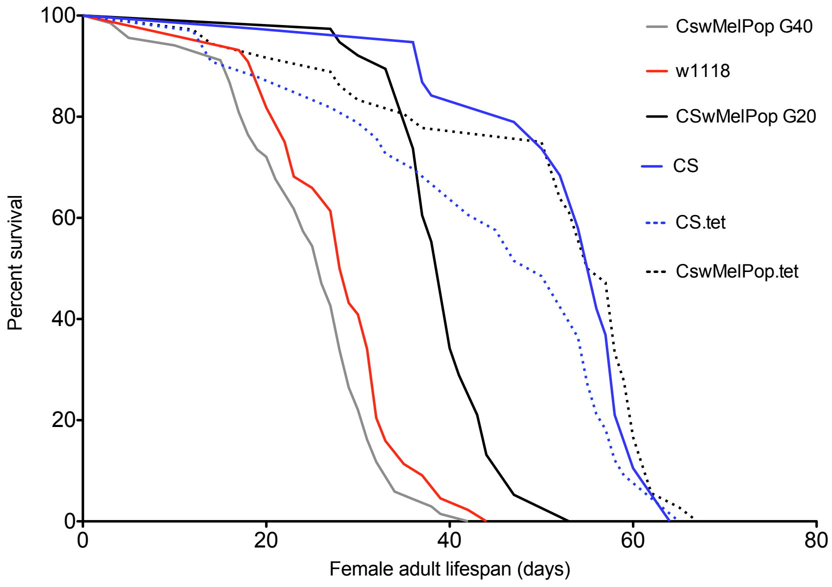

b

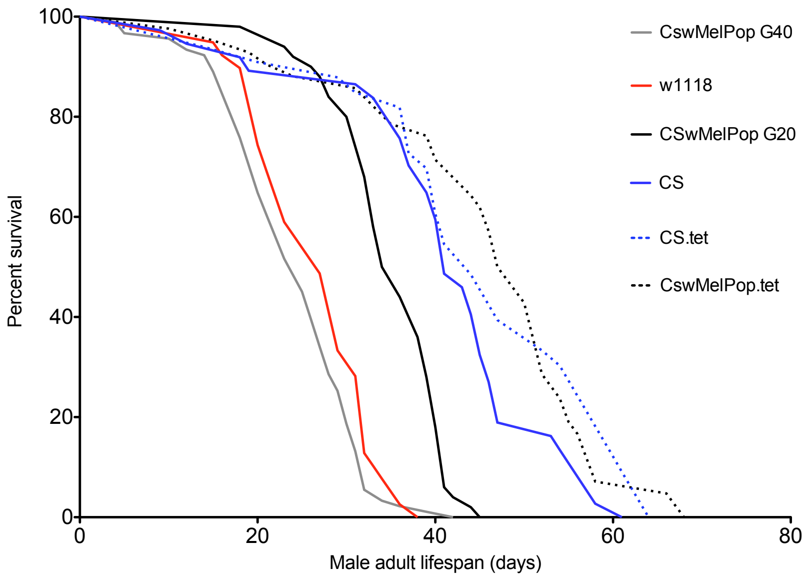

Figure S7. *Wolbachia* density over the adult lifespan in female *Drosophila melanogaster* Canton-S flies. Density of the *w*MelPop and *w*MelCS strains was determined by comparing the *wsp* (*Wolbachia*): *rps17* (*D. melanogaster*) gene ratios in 6 heads and thorax/abdomens for 3,7,14 and 28-day-old adult females. The mean density (± s.e.m) of the *w*MelCS strain (log10) was significantly greater than the *w*MelPop strain in both 3-day-old heads (two-sample t-test: t=9.271, df=10, P<0.0001) and thorax/abdomens (two-sample t-test: t=10.28, df=10, P<0.0001). For 14-day-old female flies, the densities of the two *Wolbachia* strains were not significantly different in heads (two-sample t-test: t=1.541, df=10, P=0.1544) and thorax/abdomens (two-sample t-test: t=0.741, df=10, P=0.4758). The density of the *w*MelPop strain was approximately 10 fold higher than the *w*MelCS strain in 28-day-old heads (two-sample t-test: t=6.692, df=10, P<0.0001) and approximately 7 fold higher in 28-day-old thorax/abdomens (two-sample t-test: t=8.262, df=10, P<0.0001).

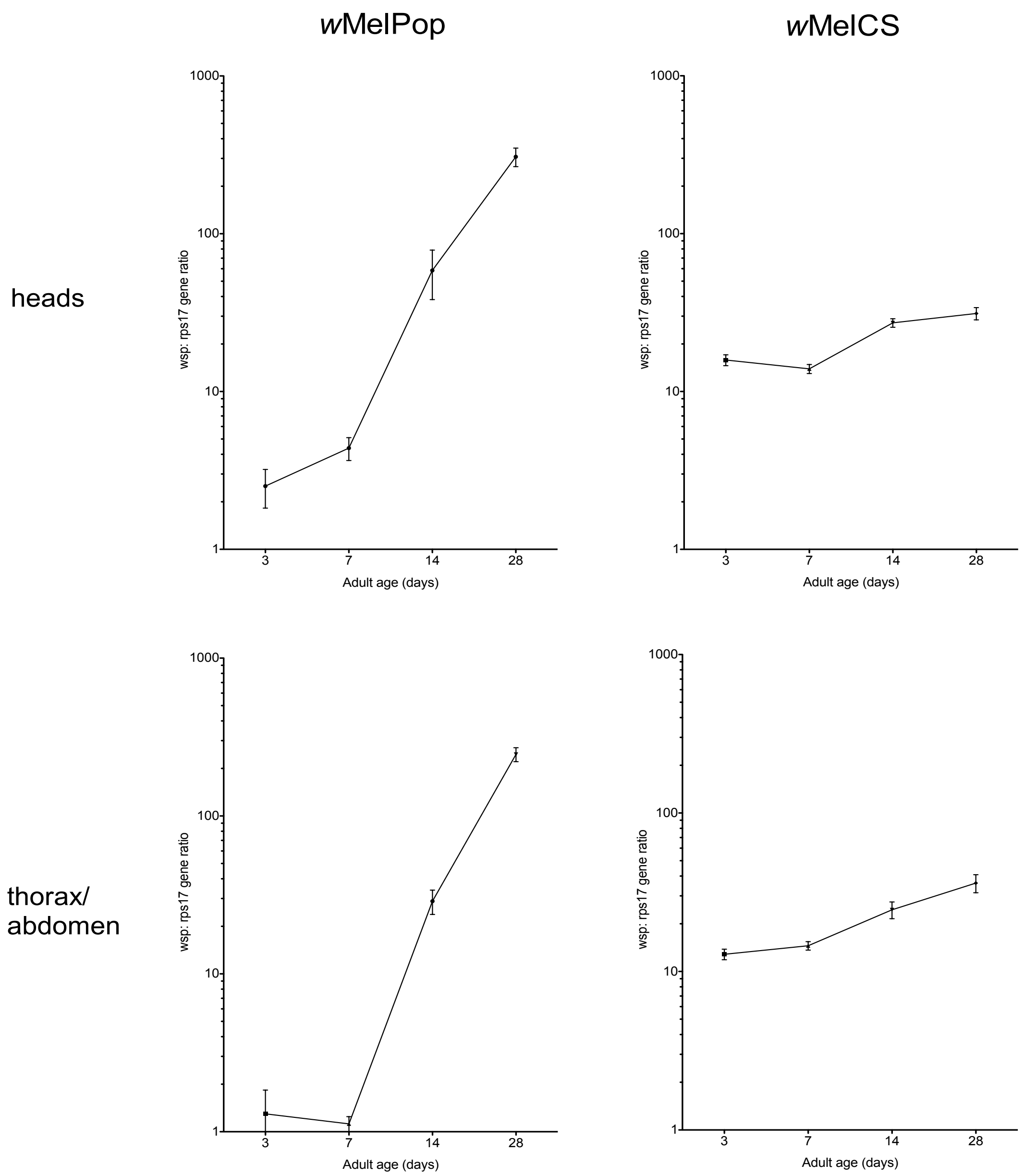

wMelPop
wMelCS
heads
thorax/
abdomen
wsp: rps17 gene ratio
1000
100
10
1
3
7
14
28
Adult age (days)

Supplementary Table 1. Primers referred to in the text

Primer	Target gene	Primer sequence (5’-3’)
Copy number variation (Fig. 4)		
1101-1103F	WD0512	CTAATGCAAACCCATGAAACCCTGC
1101-1103R	WD0513	CCATTTATAATAGCTGGGGCTATGG
1103-1104F	WD0513	GAGAATTATCTTGATAGAGTTGTACC
1103-1104R	WD0514	CGATATTGTTTTAGAGAAAACAAAGG
1213FQPCR	WD1213	GATTGGCAAGTGAAGCTAAATGAG
1213RQPCR	WD1213	CTACACTAAAGCCAGCCTTGG
wsp-FQFluv	WD1063 (*wsp*)	ATCTTTTATAGCTGGTGGTGGT
wsp-RQAll	WD1063 (*wsp*)	GGAGTGATAGGCATATCTTCAAT
Timing of genetic changes (Fig. 6)		
P1	WD0765	AGAAATGCCGCTTTCAA
P2	WD0766	CTTTTGCGATTAGAGTTTTTACTAC
P3*	WD0514	CCATATGGAGACTGTGCCTGAGAATC
P4*	WD0514	CCTCGAGTAGAAAACCTCCTGAAAAATC
P5	WD0413	TGCTACAAGACTCACACG
P6ˆ	WD0413	GCTATAAAATTTTTCATTCAATAACCTTCAA

* P3 and P4 primers were used previously for cloning purposes and contain 5’-*Nde*I and *Xho*I recognition sequences, respectively (underlined).

ˆ P6 primer contains the 10bp sequence deleted in WD0413 from *w*MelPop-CLA (underlined), therefore this primer will only amplify from DNA that does not has the deletion.

Accession numbers

*w*MelPop

BioProject: PRJNA196671
BioSample: SAMN02296948
Taxon ID: 1317678
Locus Tag Prefix: WMELPOP

Draft genome assembly:
NCBI WGS: AQQE00000000

454 reads:
SRA Submission: wMelPop-454PE
SRA Study: SRP028309
SRA Sample: SRS465572
SRA Experiment: SRX329011
SRA Run: SRR944622

Illumina reads:
SRA Submission: wMelPop-Illumina
SRA Study: SRP28309
SRA Sample: SRS465572
SRA Experiment: SRX361114
SRA Run: SRR1004280

*w*MelPop-PGYP

BioProject: PRJNA213650
BioSample: SAMN02298017
Taxon ID: 1379789

Draft genome assembly:
NCBI WGS: AQQE00000000

454 reads:
SRA Submission: wMelPop-PGYP-454PE
SRA Study: SRP028350
SRA Sample: SRS488260
SRA Experiment: SRX329944
SRA Run: SRR945786

Illumina reads:
SRA Submission: wMelPopPGYP-Illumina
SRA Study: SRP28350
SRA Sample: SRS488260
SRA Experiment: SRX361115
SRA Run: SRR1004281

*w*MelPop-CLA

BioProject: PRJNA213653
BioSample: SAMN02296993
Taxon ID: 1379790

454 reads:
SRA Submission: wMelPopCLA-454SE
SRA Study: SRP028311
SRA Sample: SRS465570
SRA Experiment: SRX329014
SRA Run: SRR944623

*w*MelCS

BioProject: PRJNA213657
BioSample: SAMN02296995
Taxon ID: 1379791

Illumina reads:
SRA Submission: wMelCS
SRA Study: SRP028313
SRA Sample: SRS465571
SRA Experiment: SRX329016
SRA Run: SRR945468

References

1. Walker T, Johnson PH, Moreira LA, Iturbe-Ormaetxe I, Frentiu FD, McMeniman CJ, Leong YS, Dong Y, Axford J, Kriesner P *et al*: **The wMel Wolbachia strain blocks dengue and invades caged Aedes aegypti populations**. *Nature*, **476**(7361):450-453.
2. O'Neill SL, Giordano R, Colbert AM, Karr TL, Robertson HM: **16S rRNA phylogenetic analysis of the bacterial endosymbionts associated with cytoplasmic incompatibility in insects**. *Proc Natl Acad Sci U S A* 1992, **89**(7):2699-2702.
3. Riegler M, Sidhu M, Miller WJ, O'Neill SL: **Evidence for a global Wolbachia replacement in Drosophila melanogaster**. *Curr Biol* 2005, **15**(15):1428-1433.
4. McMeniman CJ, Lane AM, Fong AW, Voronin DA, Iturbe-Ormaetxe I, Yamada R, McGraw EA, O'Neill SL: **Host adaptation of a Wolbachia strain after long-term serial passage in mosquito cell lines**. *Appl Environ Microbiol* 2008, **74**(22):6963-6969.
5. Osborne SE, Leong YS, O'Neill SL, Johnson KN: **Variation in antiviral protection mediated by different Wolbachia strains in Drosophila simulans**. *PLoS Pathog* 2009, **5**(11):e1000656.
